# pH-Dependent Capping Interactions Induce Large-Scale Structural Transitions in i-Motifs

**DOI:** 10.1021/jacs.2c13043

**Published:** 2023-02-06

**Authors:** Israel Serrano-Chacón, Bartomeu Mir, Lorenzo Cupellini, Francesco Colizzi, Modesto Orozco, Núria Escaja, Carlos González

**Affiliations:** † Instituto de Química Física ”Rocasolano”, CSIC, Serrano 119, 28006Madrid, Spain; ‡ Inorganic and Organic Chemistry Department, Organic Chemistry Section, and IBUB, University of Barcelona, Martí i Franquès 1-11, 08028Barcelona, Spain; § Institute for Research in Biomedicine (IRB Barcelona), The Barcelona Institute of Science and Technology (BIST), 08028Barcelona, Spain; ∥ Departament de Bioquímica i Biomedicina. Facultat de Biologia, Universitat de Barcelona, 08028Barcelona, Spain; ⊥ BIOESTRAN Associated Unit UB-CSIC, 08028Barcelona, Spain

## Abstract

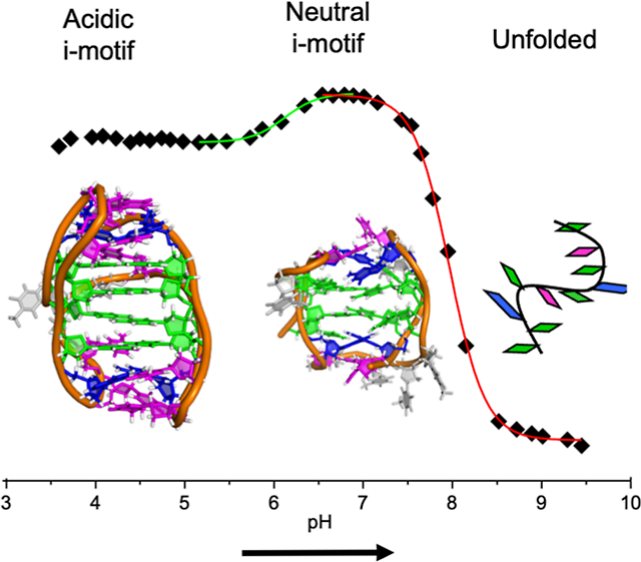

We study here a DNA oligonucleotide having the ability to form two different i-motif structures whose relative stability depends on pH and temperature. The major species at neutral pH is stabilized by two C:C^+^ base pairs capped by two minor groove G:C:G:C tetrads. The high pH and thermal stability of this structure are mainly due to the favorable effect of the minor groove tetrads on their adjacent positively charged C:C^+^ base pairs. At pH 5, we observe a more elongated i-motif structure consisting of four C:C^+^ base pairs capped by two G:T:G:T tetrads. Molecular dynamics calculations show that the conformational transition between the two structures is driven by the protonation state of key cytosines. In spite of large conformational differences, the transition between the acidic and neutral structures can occur without unfolding of the i-motif. These results represent the first case of a conformational switch between two different i-motif structures and illustrate the dramatic pH-dependent plasticity of this fascinating DNA motif.

## Introduction

Nucleic acids are dynamic and polymorphic molecules which can adopt a myriad of conformations in response to changes in the environment. Studying their conformational transitions is crucial to understand nucleic acids’ biological activity and their potential applications in bio- and nanotechnology. In particular, conformational transitions involving i-motif structures are of special relevance due to their unique pH dependence. The i-motif is a four-stranded intercalated structure stabilized by the formation of hemiprotonated C:C^+^ base pairs between parallel-oriented strands.^1−4^ Since cytosine protonation is required for their formation, i-motif structures are usually observed at acidic pH. In spite of its preference for acidic environments, recent studies have shown that the i-motif is not an exotic structure generated only under special laboratory conditions but a conformation that can be formed in the cell. The growing evidence of their formation in vivo^5,6^ and the increasing number of sequences being able to fold into stable i-motifs at neutral pH^7−11^ is arousing great attention for this structure. Moreover, it has been shown that i-motif-forming sequences are common in the genome,^10,12^ and numerous studies have described their potential role in processes like gene transcription,^13−15^ DNA synthesis,^16^ centromere^17^ and telomere^18^ maintenance, and so forth.

Besides, the strong dependency of i-motif stability on the pH makes it suitable for designing pH sensors^19,20^ or other dynamic nanodevices.^21−23^ In most cases, the conformational transitions involved in these potential devices are either i-motif-folding/unfolding transitions^19,20^ or hybridization with the complementary strand.^24^ However, the potential use of conformational switches between different i-motif structures has not been explored yet. Most studies on i-motif dynamics have focused on the folding/unfolding processes.^25−29^ Although intrinsic conformational dynamics of folded i-motif structures has been studied by computational methods,^30,31^ not many experimental studies have been performed, and most of them focused on the dynamics of loop regions.^18,32^ To the best of our knowledge, transitions between different i-motif structures have not yet been studied experimentally in atomic detail.

A significant amount of work has been focused on understanding the basis of i-motif stabilization. Factors affecting its stability include cytosine tract length,^8^ connecting loops,^11^ and capping interactions at the sides of the C:C^+^ stack.^33,34^ Among the different capping interactions, minor groove tetrads (MGTs) are of particular interest since the interaction between MGTs and the terminal C:C^+^ base pairs provides significant thermal and pH stabilization.^10,34^ MGTs are the result of the association of two base pairs through their minor groove side and have been observed with different arrangements of Watson–Crick base pairs or mismatches (e.g., A:T:A:T,^35^ A:G:A:G,^36^ G:C:G:C,^35,37,38^ G:C:G:T,^10^ or G:T:G:T^39,40^). MGTs can be “direct” or “slipped”, depending on the relative position of the two base pairs involved.^41^ In the i-motif context, all the MGTs reported to date have been slipped, forming two H(N2)(G)–N3(G) hydrogen bonds between adjacent guanines.^10,34,39,40^

The MGT/C:C^+^ interaction provides a dramatic stabilization to such an extent that i-motif forms even in sequences with very few cytosines, as those found in some repetitive sequences with no C-tracts.^10^ These repetitive sequences fold into peculiar globular i-motif structures stabilized by two hemiprotonated C:C^+^ base pairs, flanked by two MGTs. Interestingly, these structures can form in tandem and are prevalent in the human genome, being especially abundant in regulatory regions.^10^

The different base pair compositions of the MGTs may affect the conformational stability of these minimal i-motif structures. In particular, i-motifs stabilized by capping tetrads-containing G:C base pairs are very peculiar since they contain both neutral and protonated cytosines under the same experimental conditions. The simultaneous presence of C:C^+^ and G:C base pairs raises the question on the impact of pH on the stability of these structures since the stabilizing effect of G:C-containing tetrads may be disrupted at acidic conditions.

By combining nuclear magnetic resonance (NMR) spectroscopy and theoretical calculations, we determined here two i-motif structures stabilized by MGTs at two different pH conditions and explored the structural determinants of their stability. Quite surprisingly, we found a dramatic but reversible conformational change triggered by pH changes, which happens without the unfolding of the structure. This unprecedented conformational transition widens the range of pH-dependent changes associated with i-motifs and can lead to a new paradigm for pH sensors. Furthermore, it opens the range of potential i-motif structures that can be stable under physiological conditions.

## Results

Repetitive sequences containing two repeats of d(TCGTTCCGT) (**L**), d(TCGTTTCGT) (**M**), and d(CCGTTCCGT) (**N**) have been studied. Previous studies indicate that these sequences fold into similar i-motif structures regardless of the number of residues connecting the repeats, although connectors of three or four nucleotides are optimal for their stability. For this reason, we focus on sequences containing two repeats connected by four thymines (Table 1).

**Table 1:** Melting Temperatures (*T*_m_) and pH_T_ Values

name	sequence^a^	MGT	pH_T_	*T*_m_ (°C)pH 7.0	*T*_m_ (°C)pH 6.0	*T*_m_ (°C)pH 5.0
**LL4**	d(L-T_4_-L)	G:C:G:T	7.9	28.4	44.3	50.3
**MM4**	d(M-T_4_-M)	G:T:G:T	6.7	16.6	29.5	38.0
**NN4**	d(N-T_4_-N)	G:C:G:C	8.0	27.8	43.4^b^	53.3

a **L**: d(TCGTTCCGT); **M**: d(TCGTTTCGT); and **N**: d(CCGTTCCGT).

b Apparent *T*_m_ corresponding to the melting of two coexisting species.

### Thermal Stability versus pH

UV-monitored thermal denaturation experiments were recorded at different pHs. As expected for structures belonging to the i-motif family, *T*_m_ values are higher at acidic pH (see Table 1). In all the conditions tested, **MM4** is less stable than **LL4** and **NN4**, in agreement with a higher stability of GC-containing tetrads versus pure G:T:G:T tetrads. However, **LL4** and **NN4** exhibit comparable *T*_m_ values at neutral pH, suggesting that G:C:G:C and G:C:G:T tetrads confer similar stability.

### Changes in Circular Dichroism and NMR Spectra with pH

**Figure 1:**
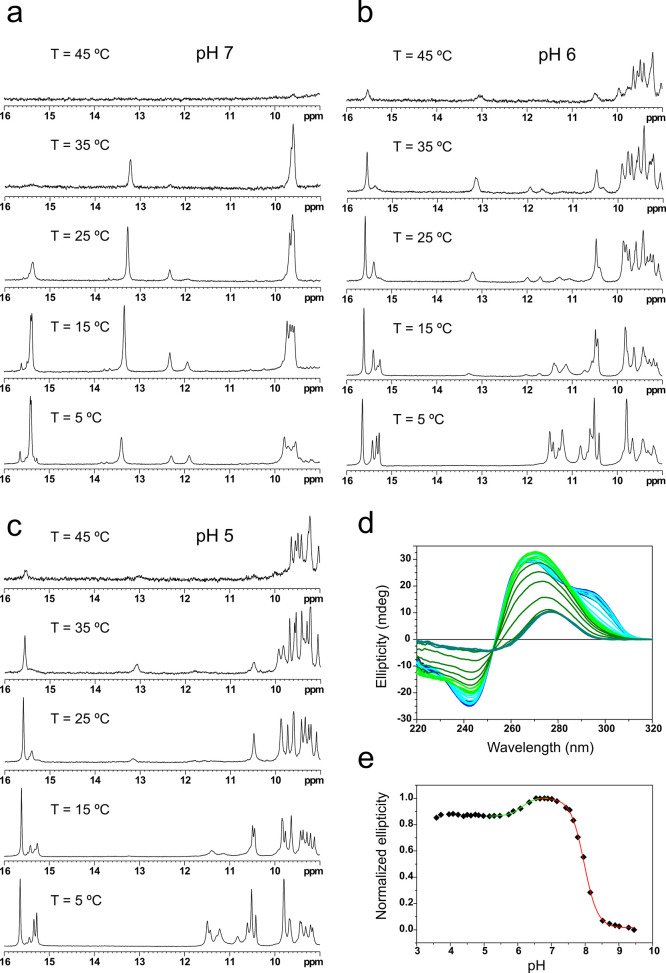
NMR spectra of **NN4** at different temperatures at pH 7 (a), 6 (b), and 5 (c). CD spectra at different pH values (from blue to green, acidic-to-basic pH) (d). Ellipticity versus pH profile (e); the two pH transitions are indicated with green (pH_T_ 6.1) and red (pH_T_ 8.0) lines. [DNA] = 500 μM (NMR) or 2 μM (CD).

Circular dichroism (CD) and NMR spectra of **NN4** at acidic pH differ from those obtained at neutral conditions, although they are in all cases consistent with i-motif folding (Figure 1a–d). Especially significant is the presence of imino signals around 15 ppm, clearly showing the formation of C:C^+^ base pairs in the pH range from 5 to 7. At neutral conditions, fewer C:C^+^ imino signals are observed, and several additional imino signals are detected between 11.5 and 13.5 ppm, most probably due to the formation of G:C base pairs. Interestingly, some of these G:C imino signals observed at neutral pH at 5 °C can also be observed at more acidic pH but only at higher temperatures. At acidic pH, signals around 10–11.5 ppm indicate the formation of G:T base pairs.

CD spectra are also pH-dependent. While at neutral pH, CD spectra exhibit a maximum band between 260 and 270 nm and a minimum band around 240 nm, at acidic conditions a second maximum is observed at around 295 nm. At basic pH, band intensities are reduced, and the CD spectrum contains a contribution from the statistical coil (see Figure 1d).

The midpoint of the pH denaturation transition (pH_T_) was estimated by following the maximum at 270 nm of CD spectra versus pH (see Figures 1e and S1 and S2). In all cases, structures exhibit very high pH_T_ values, being significantly higher for **LL4** and **NN4** than for **MM4**. This indicates that G:C-containing tetrads provide enhanced pH stability compared to G:T:G:T tetrads. The case of **NN4** is of particular relevance since the acid–base titration curve clearly exhibits two transitions with pH_T_ values of 6.1 and 8.0 (Figure 1e).

CD and NMR data are consistent with the formation of two different structures for **NN4**; their relative populations are dependent on the pH and temperature but not on the presence of Na^+^ or K^+^ counterions (Figure S2). Observation of multiple exchangeable signals at high temperatures and intermediate pH (Figure 1b) indicates that the equilibrium between neutral and acidic species is slow on the NMR timescale.

### NMR Assignment and Structural Calculation

To get insight into this peculiar behavior, we undertook the assignment of the NMR spectra of **NN4** at neutral and acidic conditions. Substitution of cytosines by 5-methyl-cytosines at certain positions was required for a complete assignment of the NMR spectra. Full details of the assignments are given in the Supporting Information (see Figures S3–S14).

**Figure 2:**
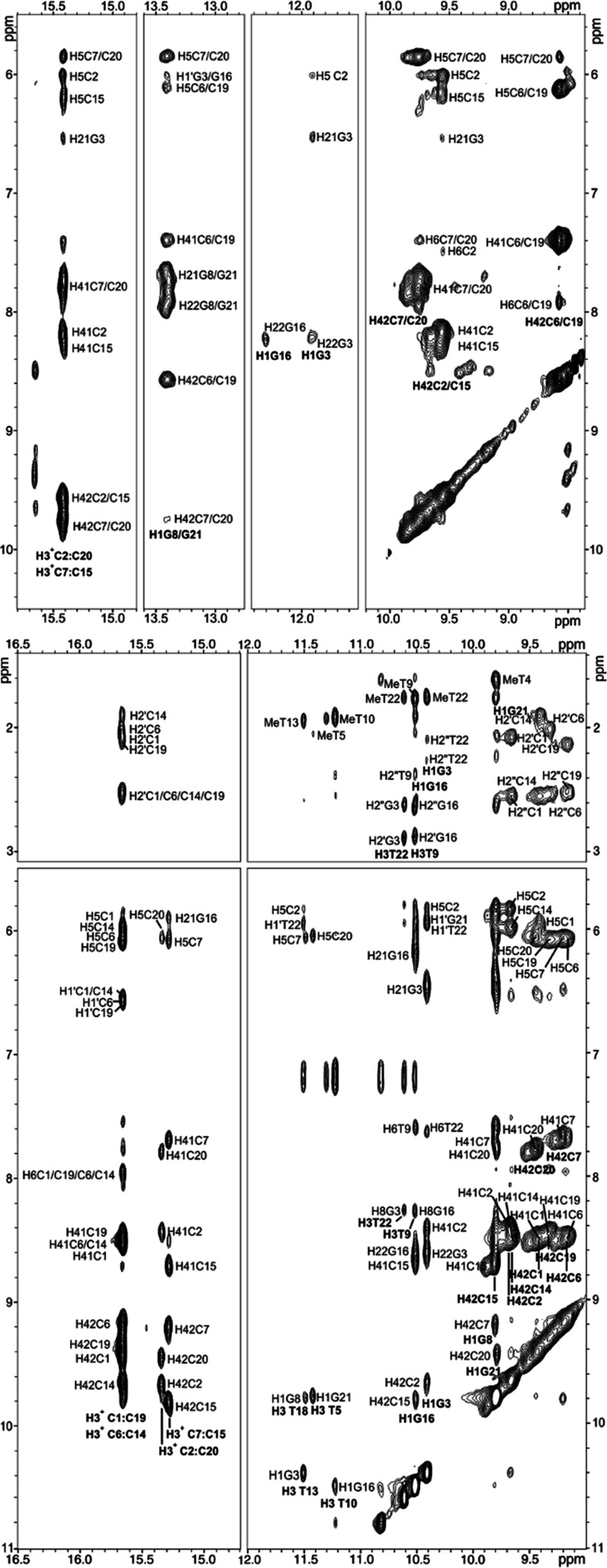
Exchangeable proton regions of NOESY spectra (150 ms mixing time) of **NN4** at pH 7 (top) and pH 5 (bottom), sodium phosphate buffer, *T* = 5 °C, and [oligonucleotide] = 500 μM.

As shown in Figure 2 (top), nuclear Overhauser enhancement spectroscopy (NOESY) spectra recorded at pH 7 show the coexistence of alternative i-motif species. One of the two imino signals from hemiprotonated C:C^+^ base pairs (15.41 ppm) corresponds to two degenerated protons of the major species, whereas the signal at 15.66 ppm, which is less intense and shows fewer contacts, belongs to the minor species. We concluded that the hemiprotonated base pairs formed at this pH are C2:C20^+^ and C7:C15^+^, showing clear H41/H42C–H3^+^C contacts. Amino protons of two cytosines (C6 and C19) exhibit cross-peaks with guanine imino protons (13.4 ppm), indicating the formation of two Watson–Crick G:C base pairs, which were assigned as G8:C19 and G21:C6. The two signals around 12 ppm were assigned to the imino protons of G3 and G16 based on a number of cross-peaks with the neighboring C:C^+^ base pairs (H5C2–H1G3). Although the chemical shifts of these imino signals might be indicative of G:C base pairs, no cross-peaks with cytosine amino protons could be detected.

Spectra of **NN4** at pH 5 exhibit more dispersed signals than those recorded at pH 7 (Figure 2, bottom). The observed minor signal at 15.66 ppm at pH 7 is more intense at pH 5, displaying a high number of cross-peaks. A comparison of signal intensities in this region suggests the overlapping of two different hemiprotonated imino signals. Two extra H3^+^ signals at 15.29 and 15.34 ppm, which also show nuclear Overhauser effect (NOE) contacts with two pairs of cytidine amino protons, confirm the formation of four C:C^+^ base pairs between nonequivalent residues. Since no imino signals are observed in the Watson–Crick region (12–14 ppm), we can conclude that those cytosines involved in the formation of G:C:G:C tetrads at neutral pH are forming C:C^+^ base pairs at acidic conditions. The stacking order of C:C^+^ base pairs could be determined on the basis of base–base NOEs (H5C–H1G) and contacts through the i-motif minor (H1′–H1′) and major grooves (H42C1:C14–H2′/H2″C14:C1 and H42C6:C19–H2′/H2″C19:C6). All this information is consistent with the formation of a four-step stack of hemiprotonated C:C^+^ base pairs with the order C2:C20^+^, C6:C14^+^, C1:C19^+^, and C7:C15^+^. Note that the cytosines forming the tetrads in the neutral structure occupy the central position of the C:C^+^ stack in the acidic one. Interestingly, four imino protons belonging to thymine residues between 11.0 and 11.5 ppm show NOE cross-peaks with imino protons of guanine residues in the 10.0 to 11.0 ppm region, indicating the formation of four G:T base pairs (involving T5, T10, T13, and T18). In addition, several H1′–H22/H1 contacts between different guanine residues indicate that these G:T base pairs are forming two G:T:G:T MGTs.

The three-dimensional structures were calculated on the basis of NMR data by following the protocols described in the Experimental Methods section. A total of 189 and 151 interproton distance constraints were used in the structural calculation of the acidic and neutral structures, respectively. Statistical analysis of the final structures including the number of distance and angular constraints are shown in Table S5. In both cases, the structures are overall well defined with total residual distance violations of 1.64 and 2.16 Å, respectively. Structural ensembles are shown in Figures S15 and S16. Overall, both structures are well defined with root-mean-square deviations (RMSDs) below 1 Å excluding some flexible residues in the loops.

### Description of the Structures

**Figure 3:**
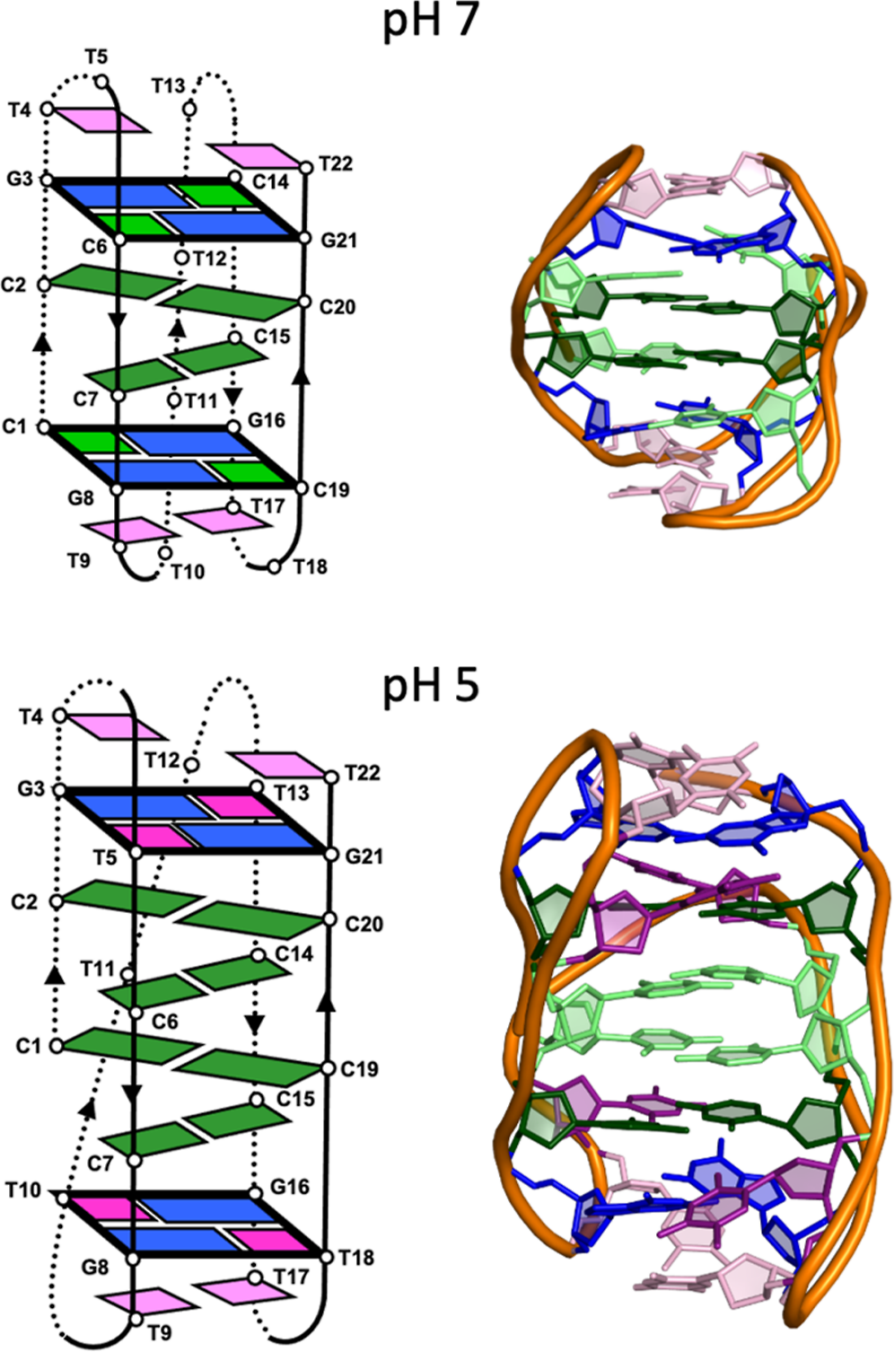
Schemes and average structures of **NN4** at neutral (top) and acidic pH (bottom). Color code: cytosines are in green, guanines are in blue, thymines involved in the tetrads are in magenta, and well-defined capping thymines are in pink. Cytosines involved in the G:C:G:C tetrad in the neutral structure are shown in lighter green. PDB codes: 8BV6 (top) and 8BQY (bottom).

The neutral structure of **NN4** consists of two C:C^+^ base pairs surrounded by two minor groove G:C:G:C tetrads (Figure 3, top). In each tetrad, one of the two GC base pairs (C1:G16 and C14:G3) is distorted (see Figures S16 and S19), probably due to interactions with the axial loop. The upfield shift of G3 and G16 imino signals and the lack of NOEs with the base-paired cytosine amino protons are consistent with these distortions. In the two lateral loops, the first thymine is stacked on top of the MGT and the second one is mainly disordered. The four thymines in the long axial loop are also disordered. Interactions contributing to the stability of this structure are most probably very similar as in the case of **LL4**. The additional stabilization expected for the extra hydrogen bond in the G:C:G:C tetrad versus G:C:G:T is not observed. This is most probably due to the high distortion in one of the G:C pairs forming the G:C:G:C tetrad. All deoxyriboses adopt a south conformation, and glycosidic angles are in anticonformation, with guanine residues in a high-anti conformation (Table S7).

The structure of the predominant species at acidic pH is formed by four C:C^+^ base pairs surrounded by two minor groove G:T:G:T tetrads, involving T5, T10, T13, and T18 (Figure 3, bottom). The two lateral loops consist of only one thymine that lies on top of the neighbor tetrad. The C:C^+^ stack, formed by C7:C15, C1:C19, C6:C14, and C2:C20, is of the 3′E type. In this structure, the axial loop consists of only two residues (T11 and T12), which are mainly disordered (see Figures S15 and S18).

Overall, the acidic structure is 5 Å more elongated than the neutral one (approximate dimensions are 23 × 15 × 8 and 18 × 15 × 7 Å for acidic and neutral structures, respectively). All sugars adopt a south conformation except for C1, C6, C14, and C19, which are in the general north domain. Glycosidic angles are all in anticonformation, with a tendency to adopt high-anti conformations (see Table S8).

### MGTs Stabilize C:C^+^ Pairs

**Figure 4:**
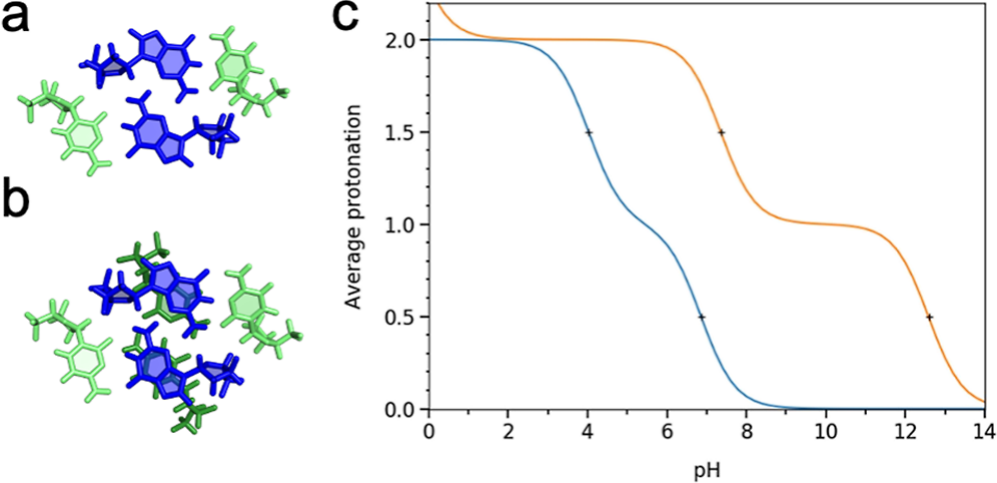
(a) MGT; (b) interaction between a C:C^+^ base pair and an MGT tetrad; and (c) calculated titration curves considering only the stack of two C:C^+^ base pairs (blue) and the same stack plus the two MGTs (orange). The black crosses indicate the titration midpoints that define the p*K*_a_.

To get more insight into the fundamental bases behind the favorable interaction between MGTs and C:C^+^ base pairs, we performed theoretical titration experiments using Poisson–Boltzmann (PB) calculations with our CMIP program^42^ (see the Experimental Section). Calculations were carried out considering the two cytosine pairs stacked with and without the capping tetrads. Results in Figure 4 clearly show a dramatic shift in p*K*_a_, provoked by the tetrads. The first calculated p*K*_a_ matches the experimentally observed midpoint of the pH denaturation transition (pH_T_) with remarkable precision. In this case, the second deprotonation event (predicted at ∼12.5) has no physical meaning since the structure is unfolded at that pH. We can conclude that the stabilization effect of MGTs in i-motifs is due to the ability of the stacked MGT to stabilize the adjacent positively charged cytosines.

### Exploring the Conformational Change Pathway

**Figure 5:**
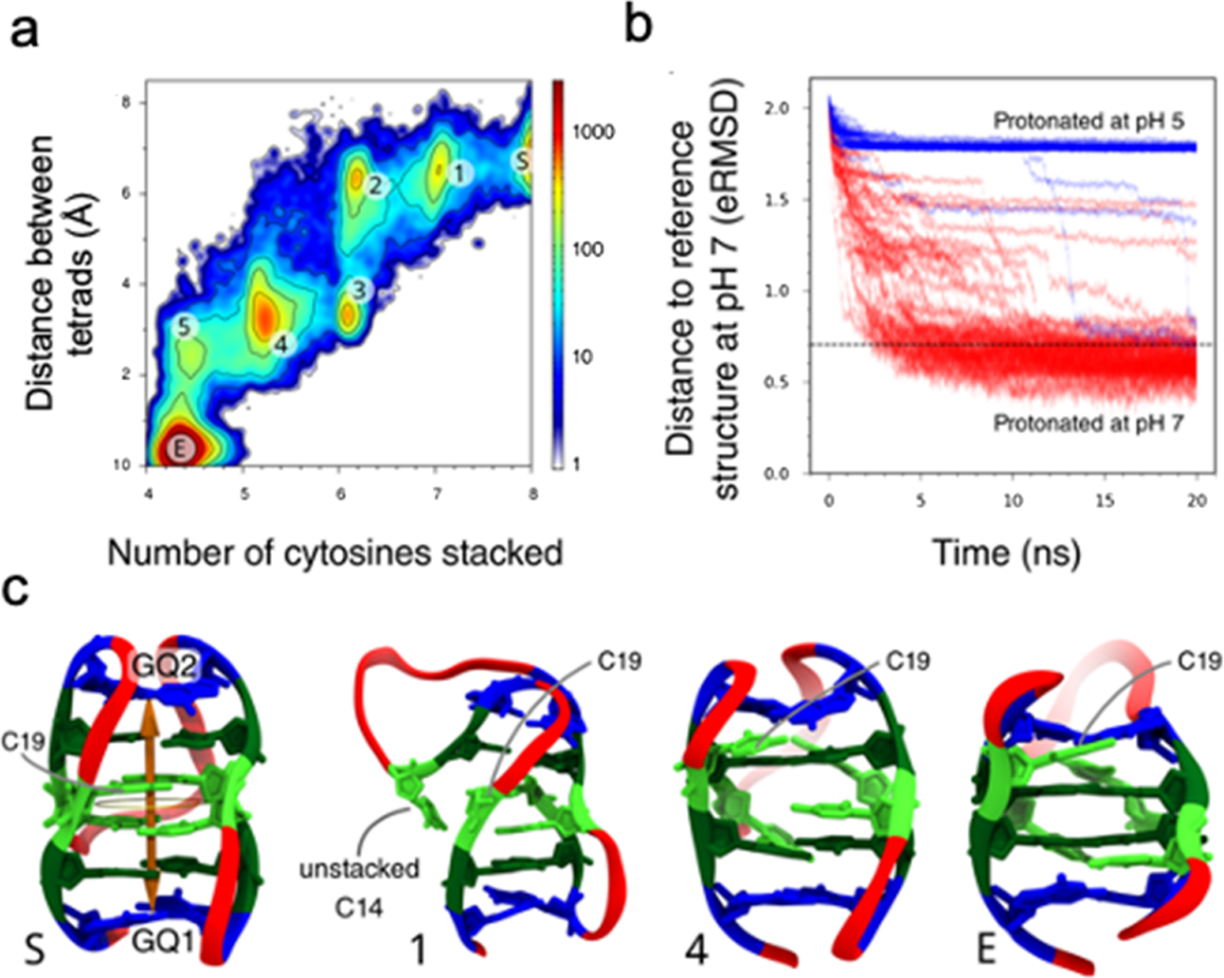
Enhanced MD results on the S (stretched, low pH) to E (exiguous, neutral pH) conformational transition. (a) Density plot of the 50 rMD trajectories (for the cytosines protonated as expected at pH 7) showing the distance between the two planes of guanines versus the number of cytosines within the stack. The color bar represents population density. The labels represent the starting (S) and end (E) states and different intermediates in the transition. (b) Distance (eRMSD) from the target structure (the neutral one) for all the rMD trajectories versus simulation time (red lines). The same data obtained from the acidic structure, in which no convergence to pH 7 structure is achieved, are also plotted. The dashed horizontal line is the eRMSD threshold value taken to be a successful transition. (c) Representation of the starting (acidic) structure, two intermediate states recognized in the density plot (a), and the final (neutral) state. Cytosines are represented in two green tones: light green for those cytosines that are neutral (C1, C6, C14, and C19) and dark green for those involved in the hemiprotonated base pairs in the neutral structure (C2, C7, C15, and C20); guanines are in blue and thymines are in red; thymine nucleobases are omitted for clarity. The double arrow represents the distance between guanine planes, whereas the ring represents the area in which cytosines are considered within the stack.

To get further insight into the conformational transition between the two structures, we used classical molecular dynamics (MD) methods. As the timescale of conformational change is substantially longer than the timescales accessible to modern computer hardware, we used ratchet-and-pawl MD (rMD)^43^ to enhance the sampling efficiency and trace the transition. Accordingly, the system is left to freely evolve along the “advance” coordinate according to thermal fluctuations, penalizing those movements that involve backward evolution along a given guess (hypothetical) reaction coordinate (information bias). In our case, the reaction coordinate is defined by the eRMSD metric taking a value of 0 when **NN4** adopts its neutral conformation (Figure 3, top). Fifty independent rMD trajectories were generated from the coordinates of the acidic structure (S in Figure 5c) but making cytosines C1, C6, C14, and C19 deprotonated as occurring at pH 7 (Figure 3, top).

Among the 50 rMD trial trajectories, 45 reached the target state within 20 ns (and the others were approaching), suggesting an easy transition that is confirmed by the free energy landscape along two intuitive coordinates: the number of stacked cytosines and the distance between the two MGTs (Figure 5a). This demonstrates that the E-state is more stable than the extended one, S. The transition occurs through a series of stable quasidegenerated intermediates which smoothly connect the S- and E-states (see Figure 5c). The first of these states corresponds to the flipping of C14 outside the stack, while the most populated intermediate (4) is a structure where only one of the deprotonated cytosines remains in the stack. Note that once the last cytosine flips, the system relaxes quickly toward the final state with a further convergence between the two tetrads. To test whether the conformational transition seen in the rMD trajectory is real and not forced by the bias, we performed a new set of 50 rMD simulations, starting from the same structure, but this time with the cytosines protonated as occurring at pH 5. These simulations, where all the hemiprotonated cytosine pairs are preserved, cannot reach the target structure (Figure 5b), strongly suggesting that there is no simulation artifact.

The above results suggest that a conformational transition from the acidic to the neutral structure can occur through flipping of four deprotonated cytosine bases and shortening of the distance between the MGTs. Remarkably, the hydrogen bonds between hemiprotonated cytosine pairs (C2:C20^+^ and C7:C15^+^) remain stable in all the rMD simulations. Illustrative examples of these rMD trajectories are shown in the Supporting Information movie. These results show that a conformational transition between the two structures can proceed without unfolding/refolding of the entire i-motif structure.

### Conformational Changes in Longer Oligonucleotides

Previous studies have shown that sequences with an increasing number of **L** repeats in tandem can fold independently adopting bead-like superstructures. Consequently, we tested whether sequences based on more than two **N** repeats can adopt similar structures and if they undergo similar transitions. An oligonucleotide consisting of four **N** repeats was studied by NMR at different pH conditions. Despite having much broader lines, the main spectral features observed for **NN4** are reproduced (Figure S20). We conclude that this sequence forms i-motif domains in tandem and that each of these domains experiences the same conformational transition between acidic and neutral pH, as illustrated in Figure S21.

## Discussion

The effect of MGTs as stabilizing capping elements in i-motif structures is now well established. However, not all possible MGTs confer the same stabilization. As described here, G:C-containing tetrads are more stabilizing than pure G:T:G:T tetrads at neutral pH. This is most probably due to the additional hydrogen bond in Watson–Crick G:C versus G:T base pairs. In the case of **NN4**, the effect is mitigated because the G:C:G:C tetrads are partially distorted; one base pair is Watson–Crick, and the other is a wobble G:C with only two H-bonds.

MGTs induce a dramatic shift in the p*K*_a_ of the cytosines involved in the neighboring C:C^+^ base pairs. Most probably, this effect is independent of the specific tetrads since all slipped MGTs have a similar geometry. In particular, the purines in the center of the tetrad are always located in the same position on top of the positively charged H3 proton, suggesting a strong PI–cation interaction.

At more acidic pH, cytosine protonation makes G:C:G:C tetrads unstable. However, G:C:G:C tetrads’ disruption does not provoke a denaturation of the compact structure but a large-scale conformational change toward an elongated structure stabilized by minor groove G:T:G:T tetrads. Its stability arises from the formation of two additional C:C^+^ base pairs, which compensate for the lower number of hydrogen bonds in G:T:G:T versus G:C:G:C tetrads. Due to the longer distance, the stabilizing effect of the tetrads on the central C:C^+^ base pairs is probably weakened. This explains the lower pH stability of this structure, with a pH_T_ between the acidic and neutral structures of 6.1 at 5 °C (Figure 1).

Interestingly, upon increasing the temperature, the neutral i-motif can be observed at a more acidic pH, as shown in the NMR spectra of Figure 1b,c. This observation reflects the lower cytosine protonation pK_a_ at higher temperatures and strongly suggests that the relative stability of the neutral and acidic i-motif structures is determined by their cytosines’ protonation states.

Not only the equilibria but also the transition between the two structures are entirely driven by the cytosines’ protonation state, as shown by our ratchet-and-pawl MD calculations. The critical role of the cytosine protonation states in i-motif dynamics concurs with recent theoretical studies in other i-motif structures.^30^ In our case, the conformational transition between the two structures involves important nucleobase rearrangements, with some residues showing dramatic rearrangements. For example, cytosines in the capping tetrads in the neutral structure occupy central positions in the i-motif core of the acidic structure. The fact that such a large conformational change may occur without the unfolding of the structure has implications for our understanding of the i-motif folding process since it indicates that, at least under certain conditions, additional C:C^+^ base pairs can be inserted in the middle of a growing C:C^+^ stack and not only in their terminal positions.

It is worth mentioning that related pH-induced conformational transitions have been observed in DNA crystals based on CGAA sequences which contain antiparallel- and parallel-stranded regions.^44^ At the junctions between the two secondary structures, some cytosines form either C:C^+^ base pairs at acidic pH or interhelical minor groove G:C:G:C tetrads at neutral conditions. Interestingly, the two variants can interconvert *in crystallo* in response to pH perturbations, giving rise to different 3D crystal lattices. These results suggest the possibility of designing dynamic biomaterial assemblies based on changes in cytosines’ protonation states.

## Conclusions

We have shown here that i-motifs may have complex dynamics. The formation of i-motif structures is not just an ON/OFF process induced by pH. Instead, some sequences can access different i-motif folds in a controlled way. Understanding these phenomena is important for applications of the i-motif in nano- and biotechnology and its potential role in the cell.

## Experimental Section

### Oligonucleotide Synthesis

Oligodeoxynucleotides were purchased at IDT with standard desalting purification. Samples were dissolved in the Na^+^ form.

### CD and UV Spectroscopy

CD spectra were recorded on a Jasco J-815 spectropolarimeter. UV spectra were recorded on a Jasco V-730 spectrophotometer. Both were fitted with Peltiers. Spectra were recorded in 25 mM sodium phosphate buffer at different pH values. Samples were initially heated at 90 °C for 5 min and slowly allowed to cool to room temperature and stored at 4 °C until use. UV melting curves were recorded at the wavelength of the larger positive band, ∼265 nm, with a heating rate of 0.5 °C·min^–1^. Uncertainties in *T*_m_ values are estimated to be ±0.2 °C.

### NMR Spectroscopy

Samples for NMR experiments were dissolved (in Na^+^ form) in either D_2_O or 9:1 H_2_O/D_2_O and 25 mM sodium phosphate buffer. The pH was adjusted by adding aliquots of concentrated HCl or NaOH. All NMR spectra were acquired on Bruker Neo Avance spectrometers operating at 600 and 800 MHz equipped with cryoprobes and processed with the TOPSPIN software. 2D NMR experiments for spectral assignment and acquirement of experimental constraints were recorded at *T* = 5 °C. A jump-and-return pulse sequence was employed to observe the rapidly exchanging protons in 1D H_2_O experiments. NOESY spectra in D_2_O and 9:1 H_2_O/D_2_O were acquired with mixing times of 150 and 250 ms. TOCSY (total correlation spectroscopy) spectra were recorded with the standard MLEV-17 spin-lock sequence and a mixing time of 80 ms. In most of the experiments using H_2_O, water suppression was achieved by including a WATERGATE module in the pulse sequence prior to acquisition. The spectral analysis program SPARKY was used for semiautomatic assignment of the NOESY cross-peaks and evaluation of the NOE intensities.

### NMR Constraints

Qualitative distance constraints were obtained from NOE intensities. NOEs were classified as strong, medium, or weak, and distance constraints were set accordingly to 3, 4, or 5 Å. In addition to these experimentally derived constraints, hydrogen bond constraints for the base pairs were used. Target values for distances and angles related to hydrogen bonds were set to values obtained from crystallographic data in related structures. Force constants were 20 kcal/mol·Å^2^ for experimental distance constraints and 30 kcal/mol·Å^2^ for hydrogen bond distance constraints. Due to the relatively broad line widths of the sugar proton signals, J-coupling constants were not accurately measured but roughly estimated from H1′–H2′ and H1′–H2″ DQF-COSY (double quantum-filtered correlation spectroscopy) cross-peaks. When these cross-peaks were consistent with deoxyribose conformations in south or south/east, sugar dihedral angles were constrained to the following target intervals: ν0(−36.5, −6.5°); ν1(19.8, 49.8°); ν2(−49.8, −19.8°); ν3(6.5, 36.5°); and ν4(−15.0, 15.0°), with a force constant of 25 kcal/mol·rad. This is equivalent to loosely constraining the sugar pseudorotation phase angles (Ps) between 144 and 180°.

### Structural Calculations

Structures were calculated with the program CYANA 3.0^45^ and further refined with the SANDER module of the MD package AMBER 18.^46^ The resulting CYANA structures were taken as starting points for the AMBER refinement, consisting of an annealing protocol in water, followed by trajectories of 500 ps each in which explicit solvent molecules were included and using the particle mesh Ewald method to evaluate long-range electrostatic interactions. Specific protocols for these calculations have been described elsewhere. The BSC1 force field^47^ was used to describe the DNA, and the TIP3P model was used to simulate water molecules. Hemiprotonated C:C^+^ base pairs were modeled by considering base pairs between neutral and protonated cytosine residues obtained from libraries included in the AMBER package. Analysis of the final structures was carried out with the programs MOLMOL^48^ and X3DNA.^49^ The conformation with the lowest AMBER total energy is taken as the best structure. Coordinates were deposited in the PDB data bank (codes 8BV6 and 8BQY for neutral and acidic structures, respectively).

### MD Simulations

Classical MD simulations were initially used to probe the conformational change from the pH 5 structure to the pH 7 structure. As the timescale of conformational change is substantially longer than those accessible even to modern computer hardware, we resorted to an enhanced sampling approach, namely, ratchet-and-pawl MD (rMD).^43^ All rMD simulations were carried out with GROMACS^50^ patched with PLUMED^51,52^ using the BSC1 force field.^47^ In rMD, a hypothetical, plausible reaction coordinate is chosen, and the system is left free to evolve along such a reaction coordinate according to the room-temperature thermal fluctuations. Conversely, a bias potential is applied whenever the system evolves backward along the reaction coordinate. In this way, the evolution of the system is dictated only by spontaneous thermal motion, and any motions that drive the system far from the target structure are discouraged.

As a reaction coordinate, we chose the distance connecting the structure at pH 5 to the structure of the i-motif at pH 7. This distance is measured in terms of the eRMSD metric, which has been proposed for nucleic acids as a more effective alternative to the common RMSD metric in discriminating different configurations.^53^ The eRMSD is able to finely distinguish different stacking and H-bonding patterns between nucleobases and is a vectorial analog of the contact maps commonly used in proteins.^54^

After thermalization and equilibration of the pH 5 structure (ensemble **S** in Figure 5c), 50 independent rMD trajectories were generated to investigate the intermediate states required to reach the NMR structure resolved at pH 7. Since deprotonation will be much faster than the conformational change, we deprotonated the cytosine pairs C14:C6 and C1:C19 for these calculations. rMD simulations were carried out with an adiabatic force constant of 200 kcal/mol per eRMSD unit and a target value of zero (i.e., eRMSD from the structure at pH 7 = 0). The eRMSD was computed excluding the thymine bases, except for those forming tetrads in the pH 5 starting structure.

### Theoretical pH Titration Calculations

Titration curves (Figure 4c) were calculated excluding the highly mobile thymine loop. The intrinsic p*K*_a_ (p*K*_int_) of each cytosine was computed from electrostatic PB calculations of the electrostatic interaction with the nontitratable residues and of the desolvation energy. The p*K*_a_ of cytosine in water was assumed to be 4.4. The interaction term *g*_*ij*_ between cytosines C_*i*_ and C_*j*_ was calculated by computing the energies of the pairs C_*i*_H^+^:C_*j*_, C_*i*_H^+^:C_*j*_, C_*i*_:C_*j*_ H^+^, and C_*i*_:C_*j*_. For PB calculations, the internal and external dielectric were set to 6 and 80, respectively.

The titration curve of each cytosine C_*i*_ can be computed by defining its average protonation ⟨*x*_*i*_⟩ at each value of the pH. The latter can be calculated from the Boltzmann average
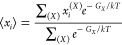
where *X* is a protonation state vector, whose elements *x*_*i*_ are either 1 or 0, depending on the protonation of site *i*. Each of these vectors represents a microstate with free energy *G*_*X*_. Given the low number of cytosines, the protonation state vectors can be all enumerated, and the sums in the equation can be computed exactly. The Gibbs free energy *G*_*X*_ can be calculated at any pH as
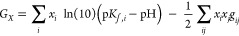
where p*K*_int,*i*_ is the aforementioned intrinsic p*K*_a_ of site *i* and *g*_*ij*_ is the interaction term between sites *i* and *j*.

The titration curve of the entire i-motif is obtained by summing all ⟨*x*_*i*_⟩. The macroscopic p*K*_a_ values of the i-motif are defined here as the midpoint of the titration curves. Unless otherwise specified, we report the p*K*_a_ value for the second protonation, given that there are two C:C stacks in the structure at pH 7.

The effect of different residues on the macroscopic p*K*_a_ was calculated by computing the titration curve after either removing the whole residue from the calculation or setting its charges to zero. In addition, we also removed the charges of the nucleobases only.

## Supporting Information Available

Detailed descriptions of NMR assignments; assignment, calculation statistics, and structural analysis; CD and NMR data; and details on the structural models (PDF)
Transition between the acidic and neutral structures (MP4)

## Abbreviations

MGT: minor groove tetrad
rMD: ratchet-and-pawl molecular dynamics
