# Supplementary material for: pH-Dependent Capping Interactions Induce Large-Scale Structural Transitions in i-Motifs
Source: J Am Chem Soc. 2023 Feb 6;145(6):3696–705. doi: 10.1021/jacs.2c13043 (PMC9936585; doi:10.1021/jacs.2c13043)
Supplement: Supplementary file 1 — ja2c13043_si_001.pdf [file ja2c13043_si_001.pdf]

ELECTRONIC SUPPORTING INFORMATION

pH-dependent capping interactions induce large-scale structural transitions in i-motifs

Israel Serrano-Chacón,[†,&] Bartomeu Mir,[†,‡] Lorenzo Cupellini,[&] Francesco Colizzi,[&] Modesto Orozco,[&,%,*] Núria Escaja[‡,§*] and Carlos González[†,§*].

† Instituto de Química Física 'Rocasolano'. CSIC. Serrano 119. 28006 Madrid. Spain

‡ Inorganic and Organic Chemistry Department. Organic Chemistry Section. and IBUB. University of Barcelona. Martí i Franquès 1-11. 08028 Barcelona. Spain

[&] Institute for Research in Biomedicine (IRB Barcelona). The Barcelona Institute of Science and Technology (BIST). 08028 Barcelona. Spain

[%] Departament de Bioquímica i Biomedicina. Facultat de Biología. Universitat de Barcelona. 08028 Barcelona. Spain

§ BIOESTRAN associated unit UB-CSIC

Supplementary Figures

Figure S1. CD spectra at different pH values and pH_T estimation of **LL4** and **MM4**. S-2
Figure S2. NMR, CDs spectra, and pH_T estimation of **NN4** in potassium buffer. S-2
Figure S3. Comparison of TOCSY spectra of **NN4** with **NN4-M2** and **NN4-M2M15** at neutral pH. S-3
Figure S4. Exchangeable protons regions of NOESY spectra of **NN4-M2**. S-3
Figure S5. Non-exchangeable protons regions of NOESY spectra of **NN4-M2**. S-4
Figure S6. Exchangeable protons regions of NOESY spectra of **NN4-M2M15**. S-5
Figure S7. Non-exchangeable protons region of NOESY spectra of **NN4-M2M15**. S-6
Figure S8. Exchangeable protons region of NOESY spectrum of **NN4** at neutral pH. S-7
Figure S9. Comparison of TOCSY spectra of **NN4** with **NN4-M2** and **NN4-M2M15** at acidic pH. S-7
Figure S10. Exchangeable protons regions of NOESY spectra of **NN4-M2** at acidic pH. S-8
Figure S11. Non-exchangeable protons regions of NOESY spectrum of **NN4-M2** at acidic pH. S-9
Figure S12. Exchangeable protons regions of NOESY spectra of **NN4-M2M15** at acidic pH. S-10
Figure S13. Non-exchangeable protons regions of NOESY spectra of **NN4** at acidic pH. S-11
Figure S14. DQF-COSY spectra of **NN4.** S-12
Figure S15. Structure ensemble of the neutral and acidic species of **NN4.** S-13
Figure S16. Detail of the structure ensemble of the neutral species of **NN4.** S-13
Figure S17. Representative structure neutral species of **NN4**. S-14
Figure S18. Representative structure acidic species of **NN4**. S-15
Figure S19. Details of the main interactions stabilizing the structures. S-16
Figure S20. NMR spectra of oligos with multiple **NN4** repeats at different temperature and pH. S-17
Figure S21. Models of tandem repeats of **NN4** at neutral (A) and acidic pH (B). S-18

Supplementary Tables

Table S1. Chemical shift lists of **NN4** (pH 7, T=5°C). S-19
Table S2. Chemical shift lists of **NN4** (pH 5, T=5°C). S-19
Table S3. Chemical shift lists of **NN4-M2** (pH 7, T=5°C). S-20
Table S4. Chemical shift lists of **NN4-M2** (pH 5, T=5°C). S-20
Table S5. Experimental constraints and calculation statistics of **NN4.** S-21
Table S6. Deoxyribose conformations of **NN4** according to J-coupling data. S-22
Table S7. Average dihedral angles and order parameters of the structure of **NN4** at pH7. S-23
Table S8. Average dihedral angles and order parameters of the structure of **NN4** at pH5. S-24

Supplementary Figures

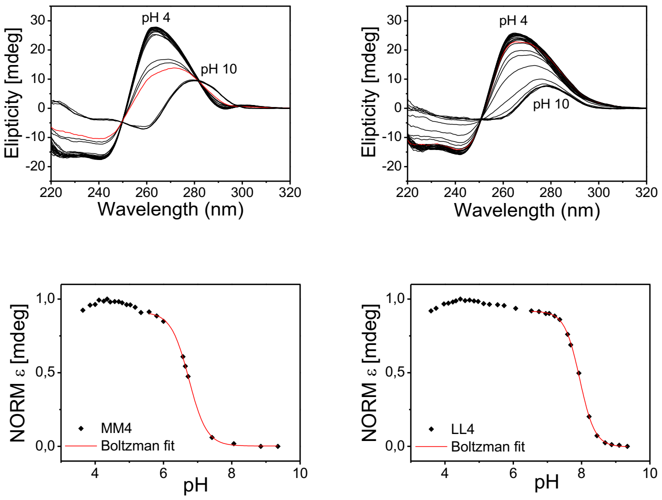

Figure S1.- Top: CD spectra superposition of **MM4** (left) and **LL4** (right) at different pH values. Spectra at neutral pH are shown in red. Bottom: CD-monitored pH titration curves of **MM4** (left) and **LL4** (right). Boltzmann fit for the denaturalization of the structures is shown in red. Experimental conditions: 25 mM sodium phosphate buffer T= 5°C. [oligonucleotide] = 2.0 μM.

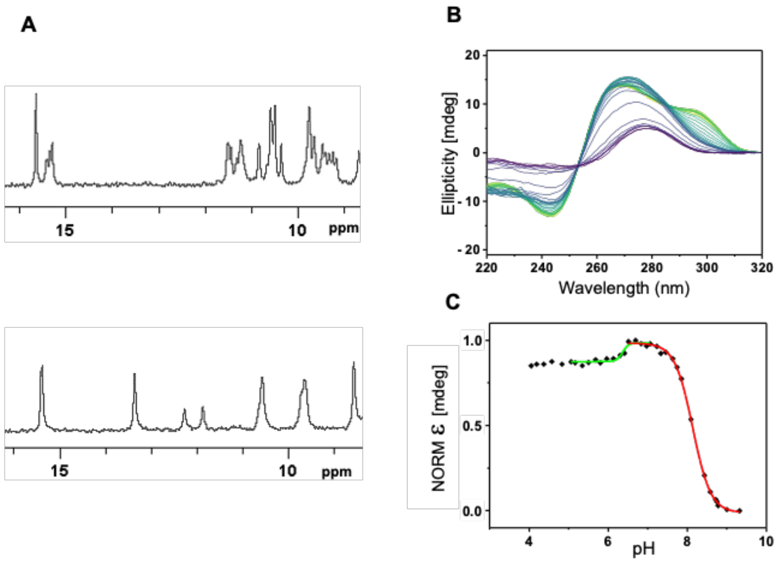

Figure S2.- A) NMR spectra of **NN4** in K+ buffer at pH 5 (top) and pH 7 (bottom) (T= 5°C). B) CD spectra superposition of **NN4** in potassium phosphate buffer. Spectra at neutral pH are shown in red. C) CD-monitored pH titration curves of **NN4**. Boltzmann fit for the denaturalization of the structures is shown in red (pH_T 8.1), and for the transition between acidic and neutral structures in green (pH_T 6.3). Experimental conditions: 25 mM potassium phosphate buffer T= 5°C. [oligonucleotide] = 2.0 μM.

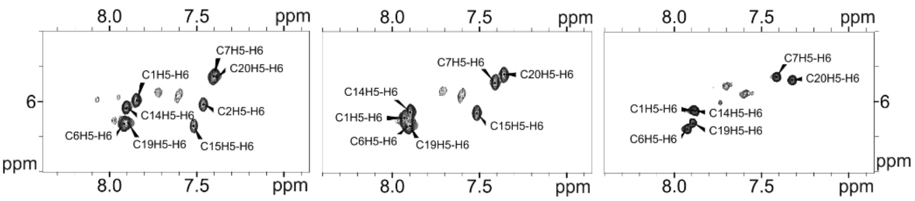

Figure S3.- From left to right. H5-H6 cross-peaks region of TOCSY *spectra (80 ms mixing time) of* **NN4**. **NN4-M2** and **NN4-M2M15**. Comparison of H5-H6 cross-peaks region of TOCSY spectra of **NN4** with two modified sequences in which a single cytosine in position 2 (**NN4-M2**) or two cytosines in positions 2 and 15 (**NN4-M2M15**) have been replaced by 5-methyl-cytosines. Unambiguous assignment of C2 and C15 residues was carried out by observing missing H5-H6 cross-peaks in **NN4-M2** and **NN4-M2M15** spectra. Chemical shifts of H5/H6 protons of C6/C19 and C7/C20 do not significantly change upon methylation. Experimental conditions: phosphate buffer pH 7, T=5°C, [oligonucleotide]=560 μM.

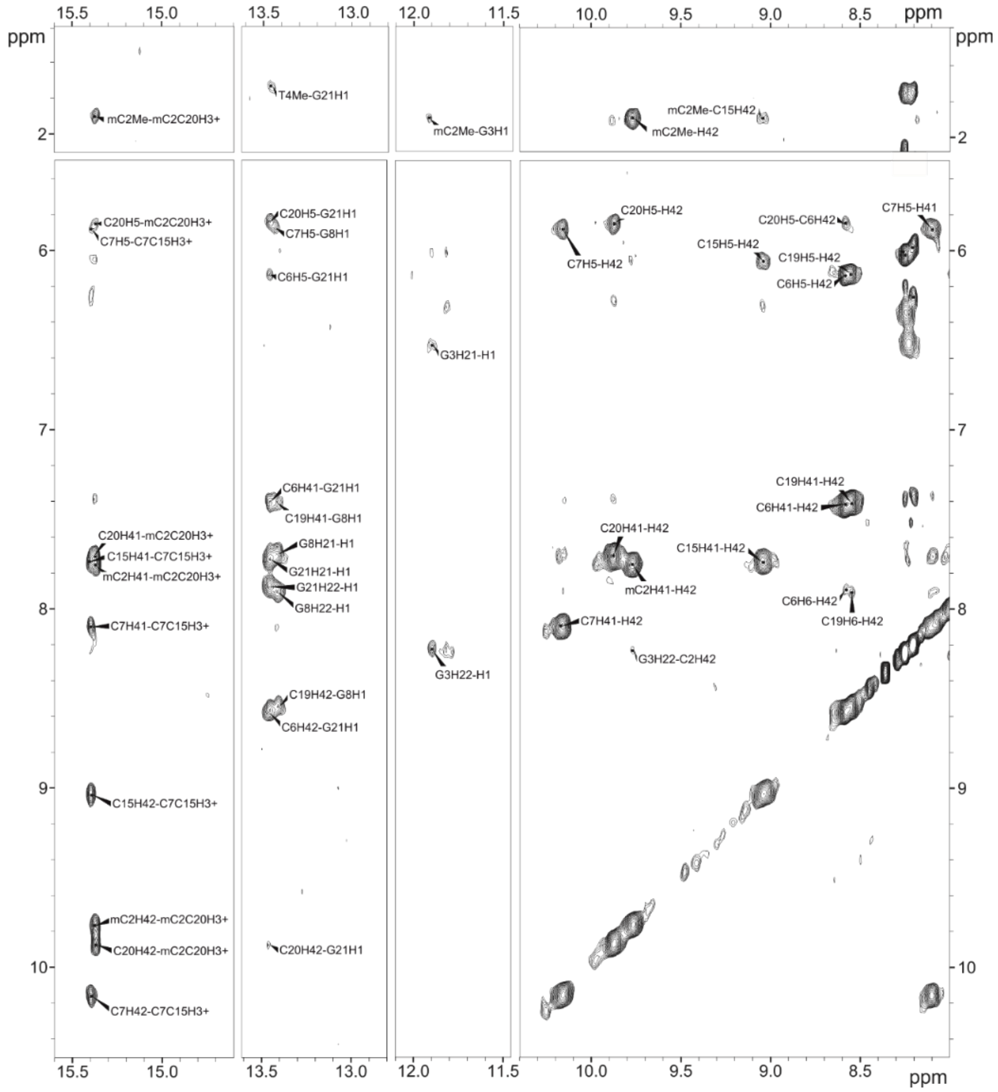

Figure S4.- Exchangeable protons regions of NOESY spectrum (150 ms mixing time) of **NN4-M2**. phosphate buffer pH 7, T=5 °C, [oligonucleotide]= 560 μM.

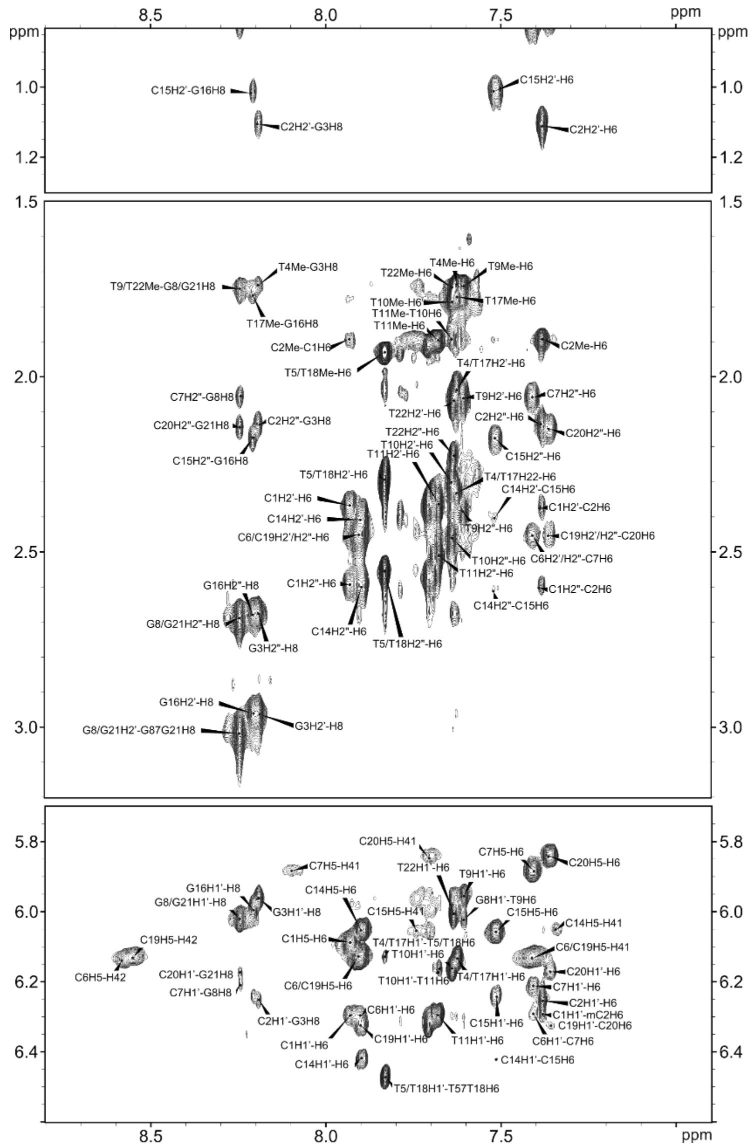

Figure S5.- Non-exchangeable protons regions of NOESY spectrum (150 ms mixing time) of **NN4-M2**, phosphate buffer pH 7, T=5 °C. [oligonucleotide]= 560 μM.

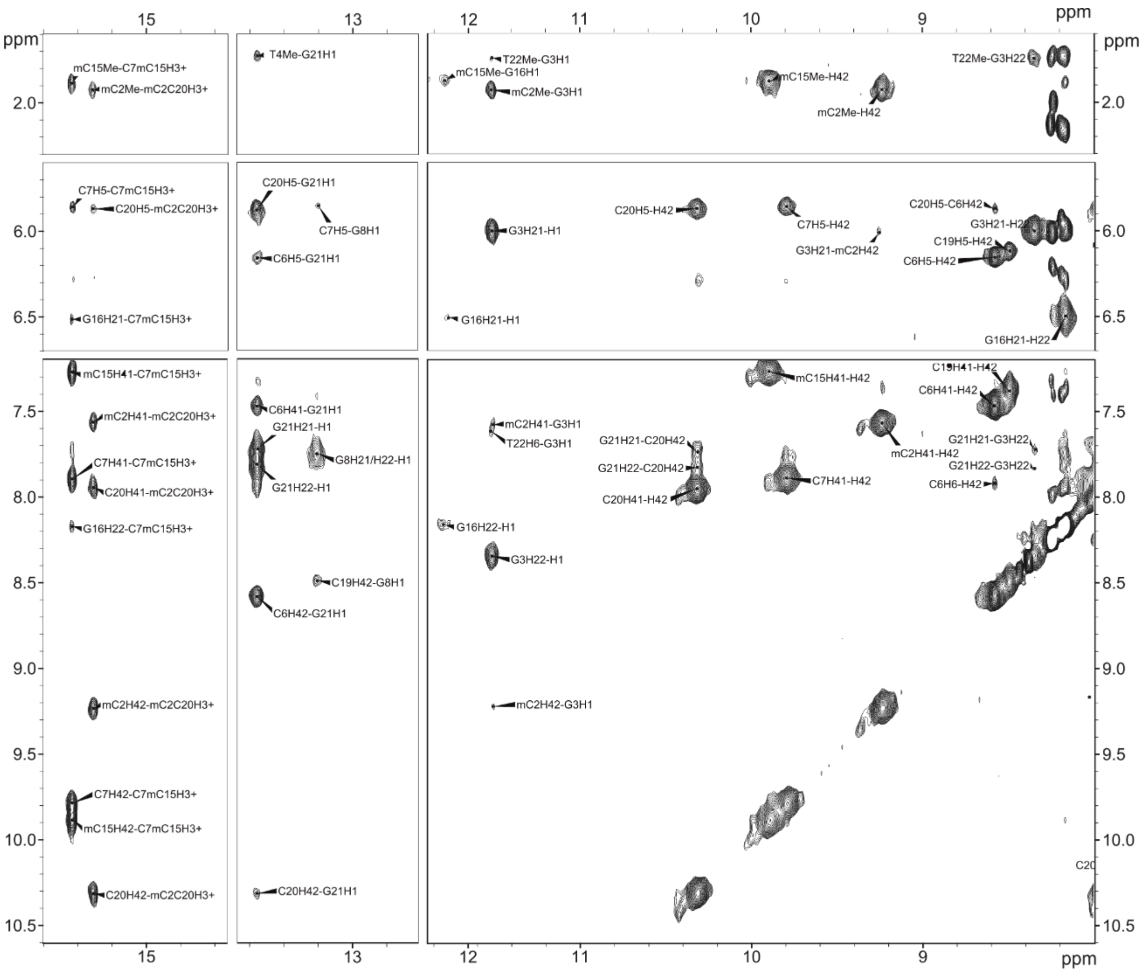

Figure S6.- Exchangeable protons regions of NOESY spectrum (150 ms mixing time) of **NN4-M2M15**, phosphate buffer pH 7, T=5 °C. [oligonucleotide]= 560 μM.

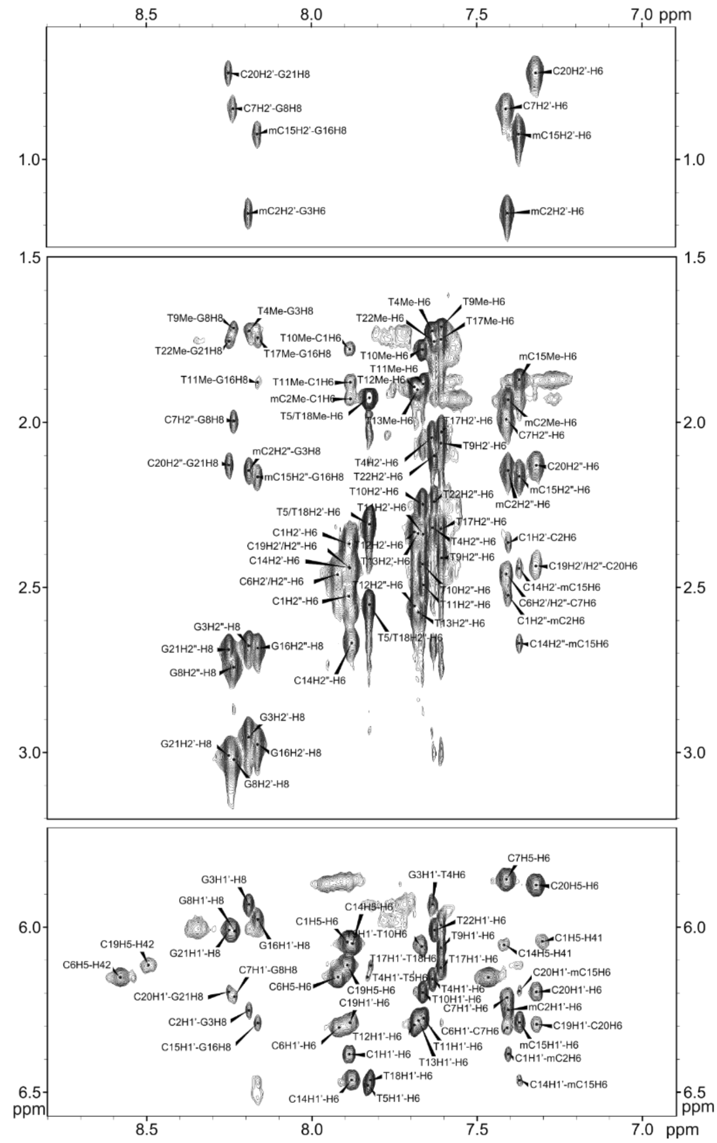

Figure S7.- Non-exchangeable protons regions of NOESY spectrum (150 ms) of **NN4-M2M15**, phosphate buffer pH 7, T=5 °C, [oligonucleotide]= 560 μM.

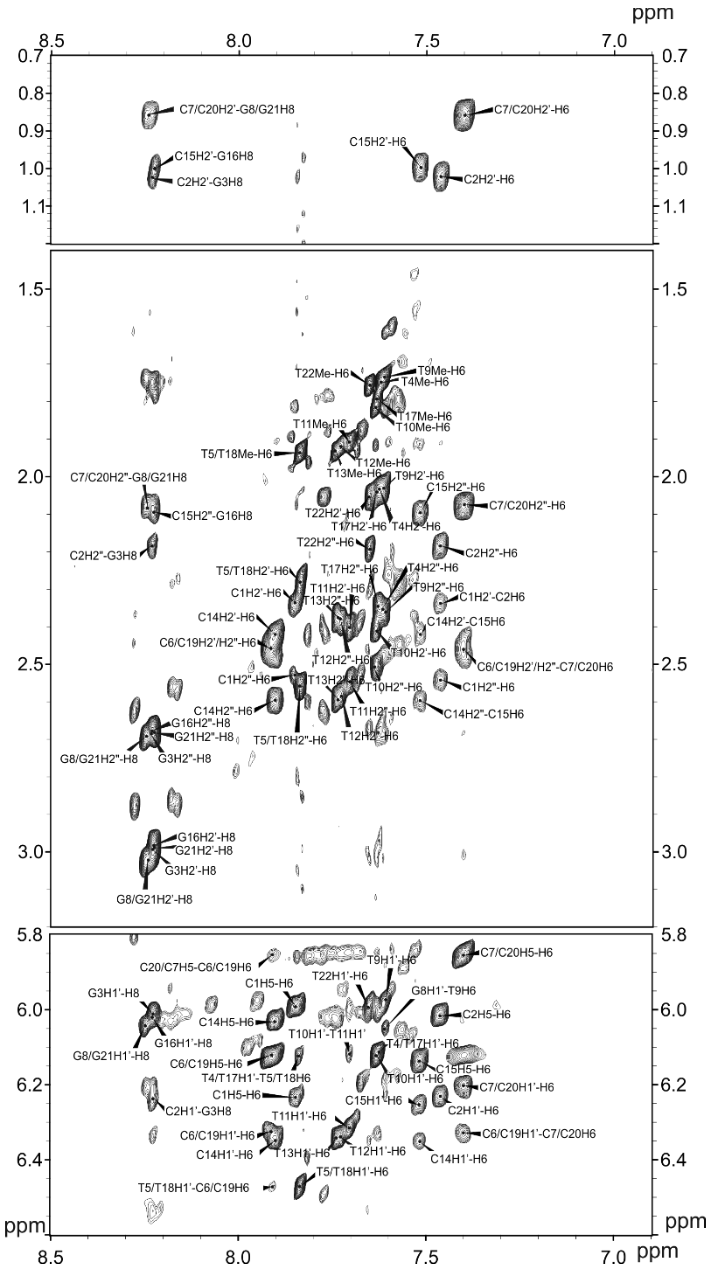

Figure S8.- Non-exchangeable protons regions of NOESY spectrum (150 ms mixing time) of **NN4**, phosphate buffer pH 7, T=5 °C, [oligonucleotide] = 560 μM.

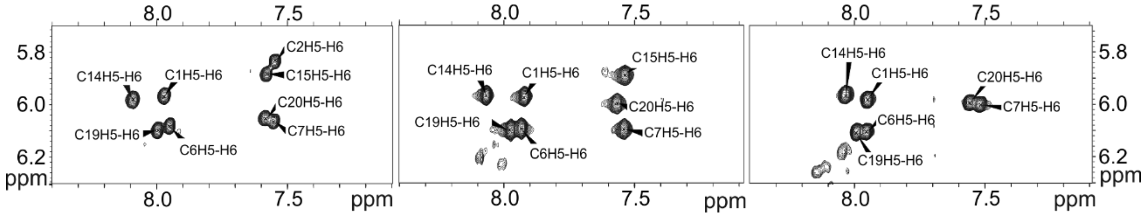

Figure S9.- From left to right. H5-H6 cross-peaks region of TOCSY spectra (80 ms mixing time) of **NN4**. **NN4-M2** and **NN4-M2M15**. Comparison of H5-H6 cross-peaks region of TOCSY spectra of **NN4** with two modified sequences in which a single cytosine in position 2 (**NN4-M2**) or two cytosines in positions 2 and 15 (**NN4-M2M15**) have been replaced by 5-methyl-cytosines. Unambiguous assignment of C2 and C15 residues was carried out by observing missing H5-H6 cross-peaks in **NN4-M2** and **NN4-M2M15** spectra. Chemical shifts of H5/H6 protons of C1. C6. C14 and C19 do not significantly change upon methylation. Experimental conditions: phosphate buffer pH 5, T=5°C, [oligonucleotide]=560 μM.

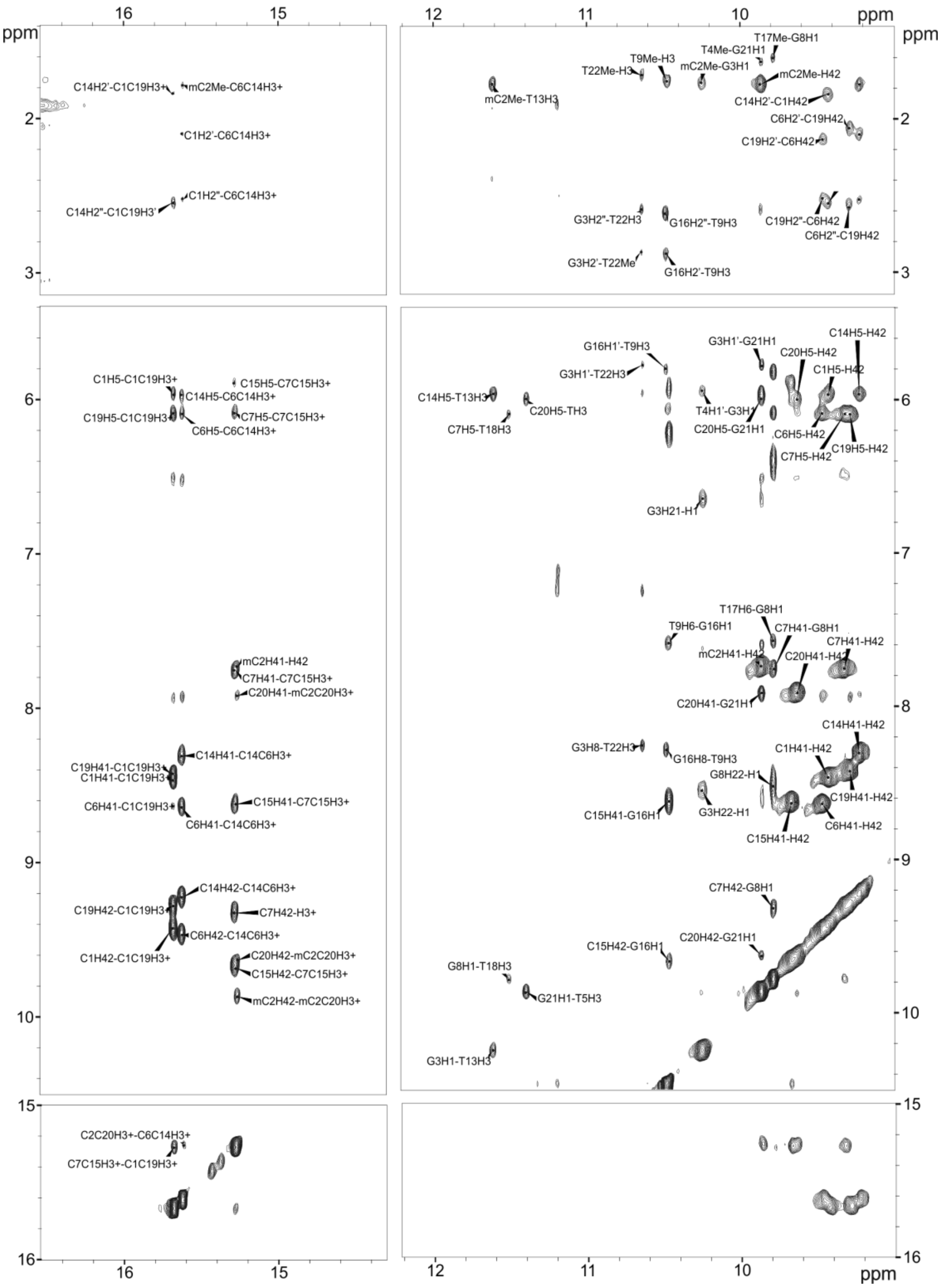

Figure S10.- Exchangeable protons regions of NOESY spectrum (150 ms mixing time) of **NN4-M2**, phosphate buffer pH 5, T=5 °C, [oligonucleotide]= 560 μM.

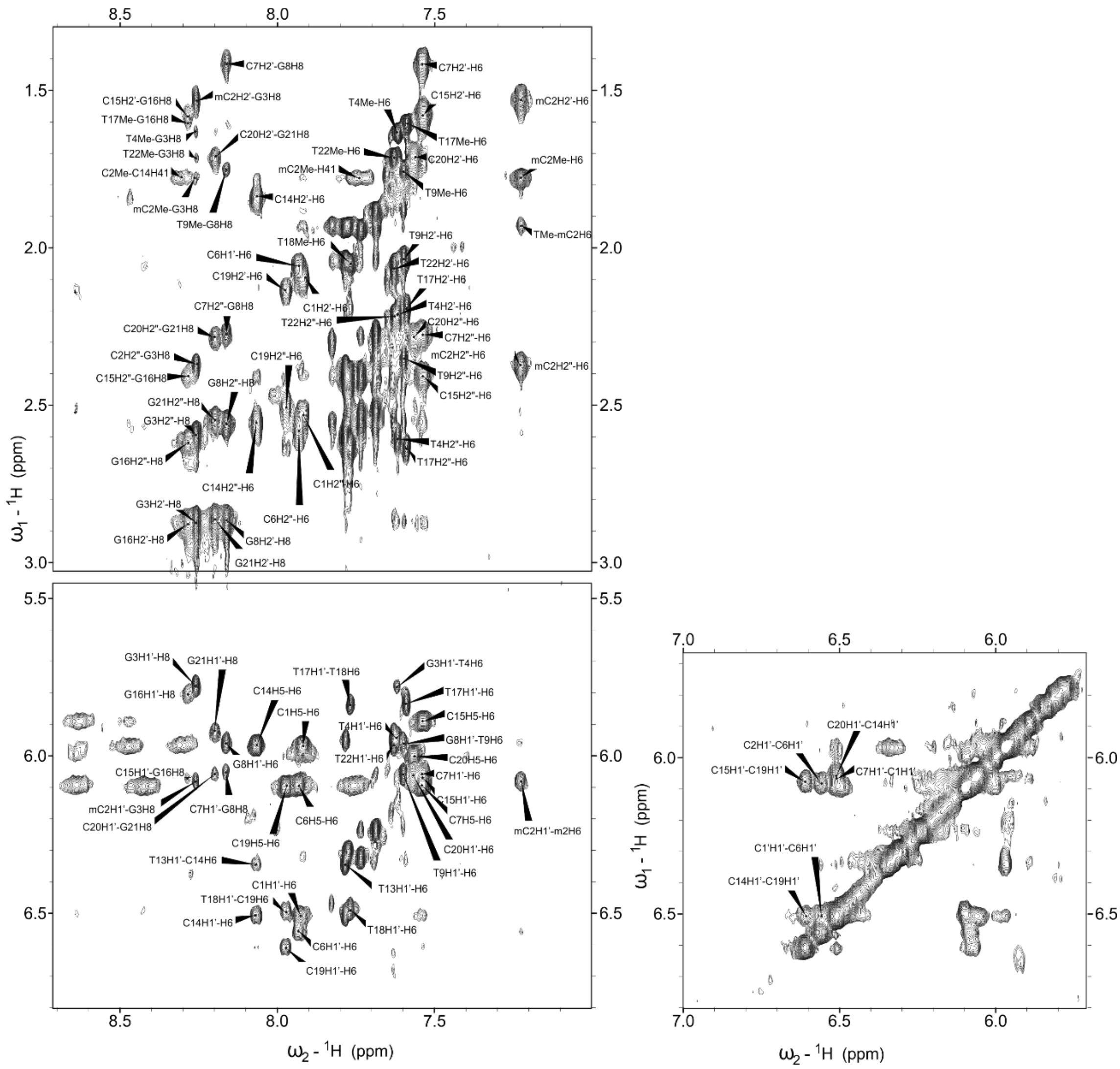

Figure S11.- Non-exchangeable protons regions of NOESY spectrum (150 ms mixing time) of **NN4-M2**, phosphate buffer pH 5, T=5 °C, [oligonucleotide]= 560 μM.

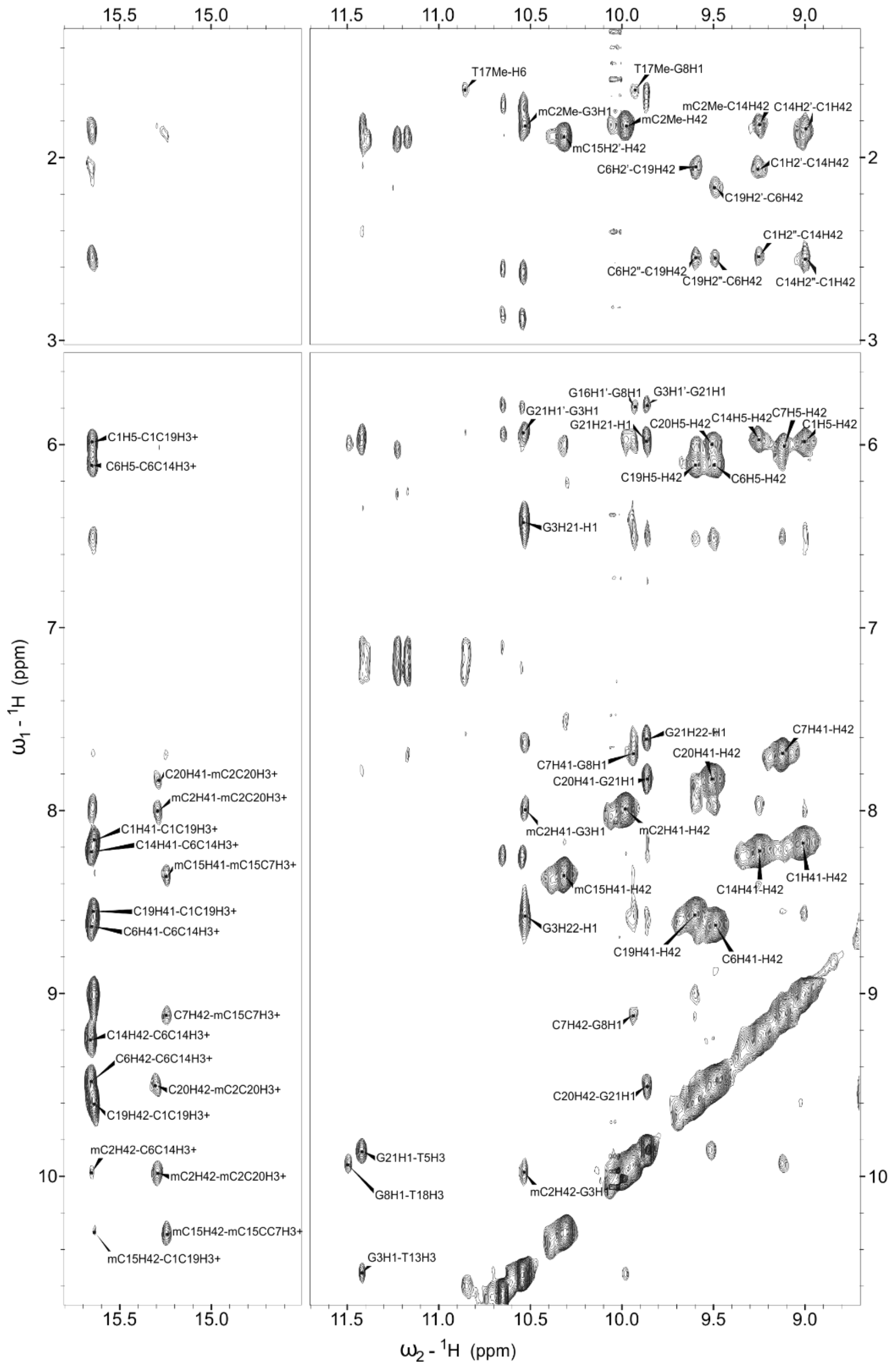

Figure S12.- Exchangeable protons regions of NOESY spectrum (150 ms mixing time) of **NN4-M2M15**, phosphate buffer, pH 5, T=5 °C, [oligonucleotide]= 560 μM.

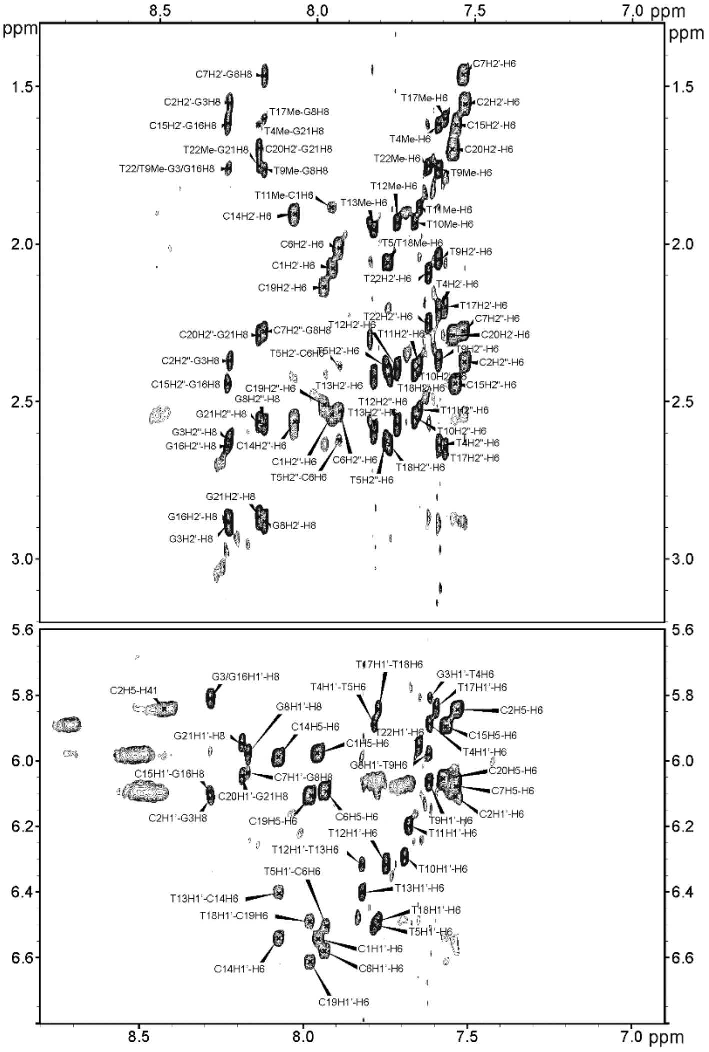

Figure S13.- Non-exchangeable protons regions of NOESY spectrum (150 ms mixing time) of **NN4**, phosphate buffer pH 5, T=5 °C, [oligonucleotide]= 560 μM.

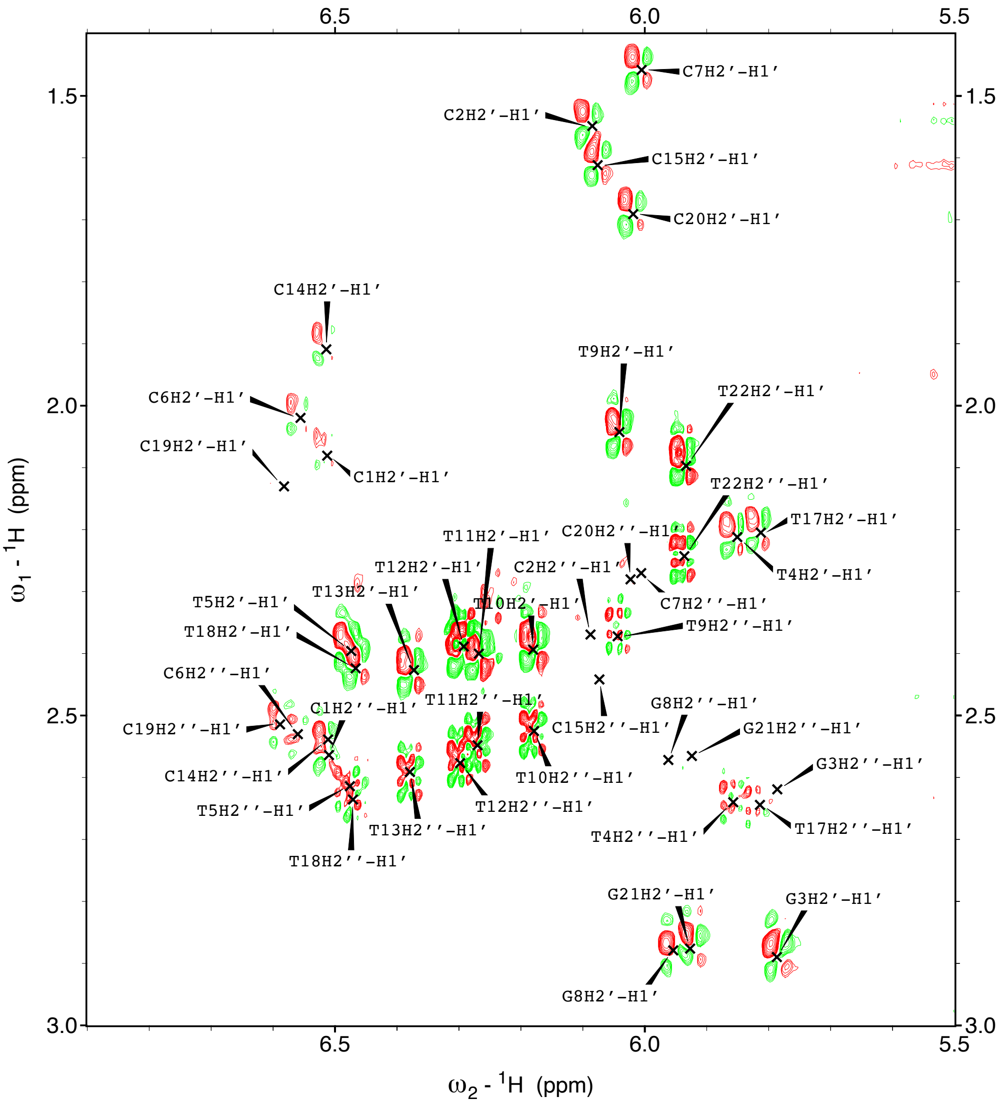

Figure S14.- H1´-H2´region of the DQF-COSY spectra of **NN4**, sodium phosphate buffer, pH 5, T=5 °C. [oligonucleotide]= 560 μM.

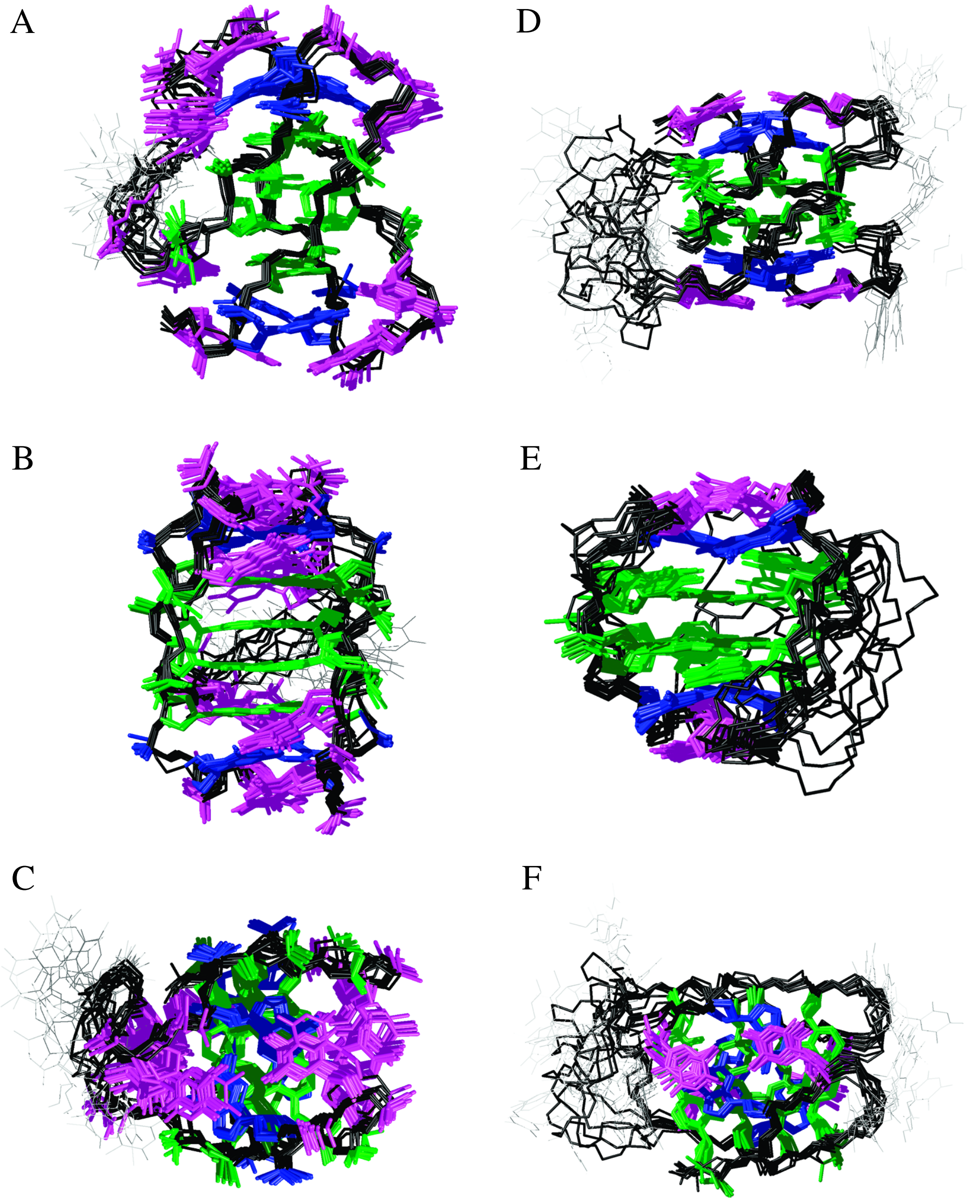

Figure S15.- Superposition of 10 refined structures of the neutral (right) and acidic (left) species of **NN4**. A) and D) View from the minor groove. B) and E) view from the major groove. C) and F) top view. Color code: Cytosines in green, guanines in blue, and well-defined thymines in magenta. Non well-defined thymines are shown in grey. Backbone is shown in black.

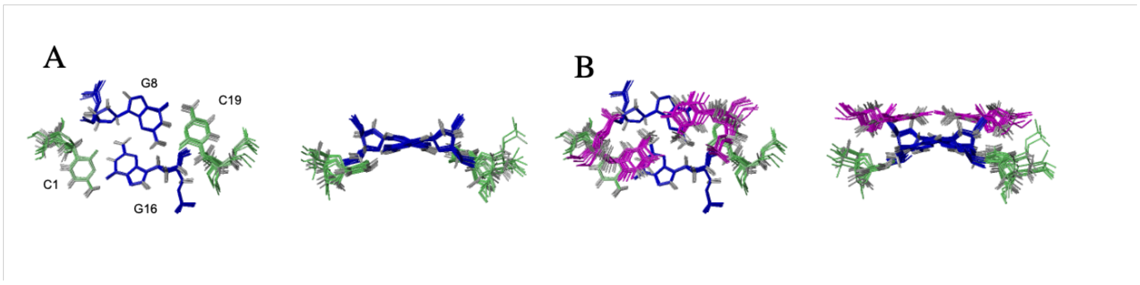

Figure S16.- Detail of the superposition of 10 refined structures of the neutral species of **NN4**. Frontal and lateral views of the G:C:G:C tetrad (A), and their interaction with the capping thymines (B). Same color code as the previous figure.

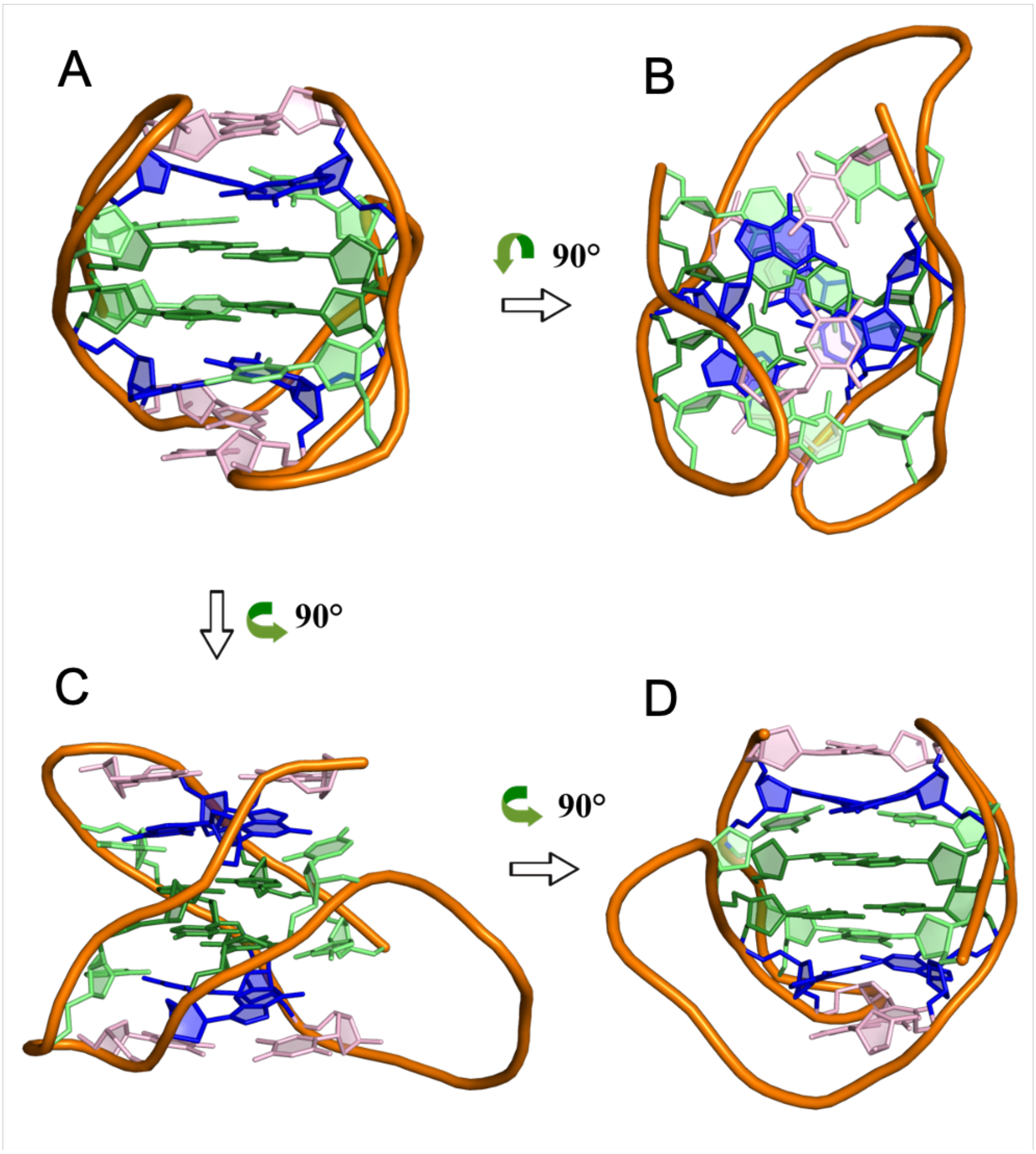

Figure S17.- Representative structure of **NN4** at neutral pH. A) View from the minor groove. B) Top view. C and D) Views from the two major grooves. Color code: Cytosines involved in C:C+ pairs in dark green, cytosines in G:C:G:C tetrads in light green, guanines in blue, and well-defined capping thymines in pink.

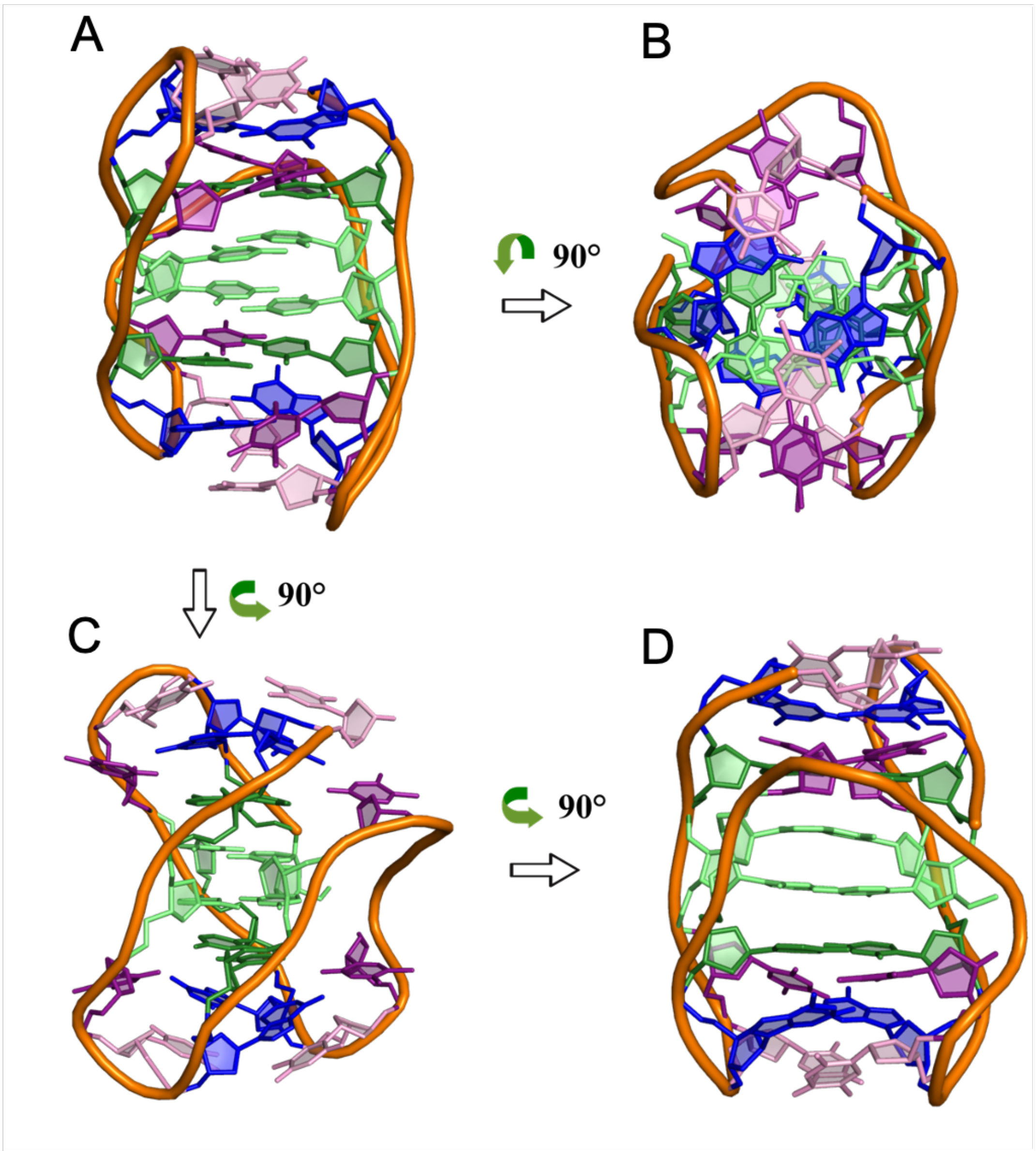

Figure S18.- Representative structure of **NN4** at acidic pH. A) View from the minor groove. B) Top view. C and D) Views from the two major grooves. Color code: Cytosines in green, guanines in blue, thymines involved in the tetrads in magenta, and well-defined capping thymines in pink. Cytosines involved in C:C+ in the neutral structure are shown in dark green, and those involved in G:C:G:C in the neutral structure are shown in light green.

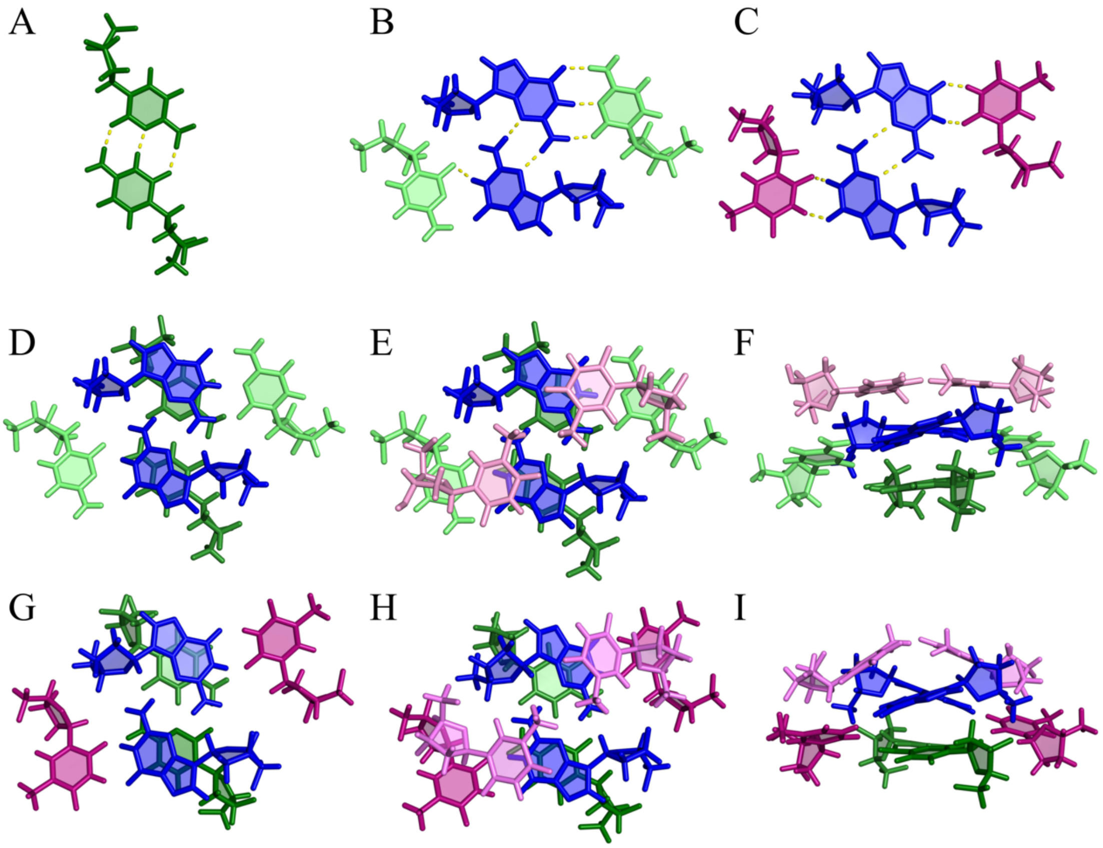

Figure S19.- Details of the main interactions stabilizing the structures. A) C:C+ base-pair; B) Minor groove G:C:G:C tetrad found in the neutral structure; C) G:T:G:T minor grove tetrad found in the acidic structure; Stacking interactions between minor groove tetrads and the nearby C:C+ base-pair in the neutral (D) and acidic (G) structures. Top (E,H) and lateral (F,I) views showing the interactions between the minor groove tetrads with C:C+ underneath and the closing thymines in the loop in the neutral (E,H) and acidic (F,I) structures. Color code: Cytosines in green (those involved in C:C+ pairs are in darker green), guanines in blue, thymines involved in the tetrads in magenta, and well-defined capping thymines in pink.

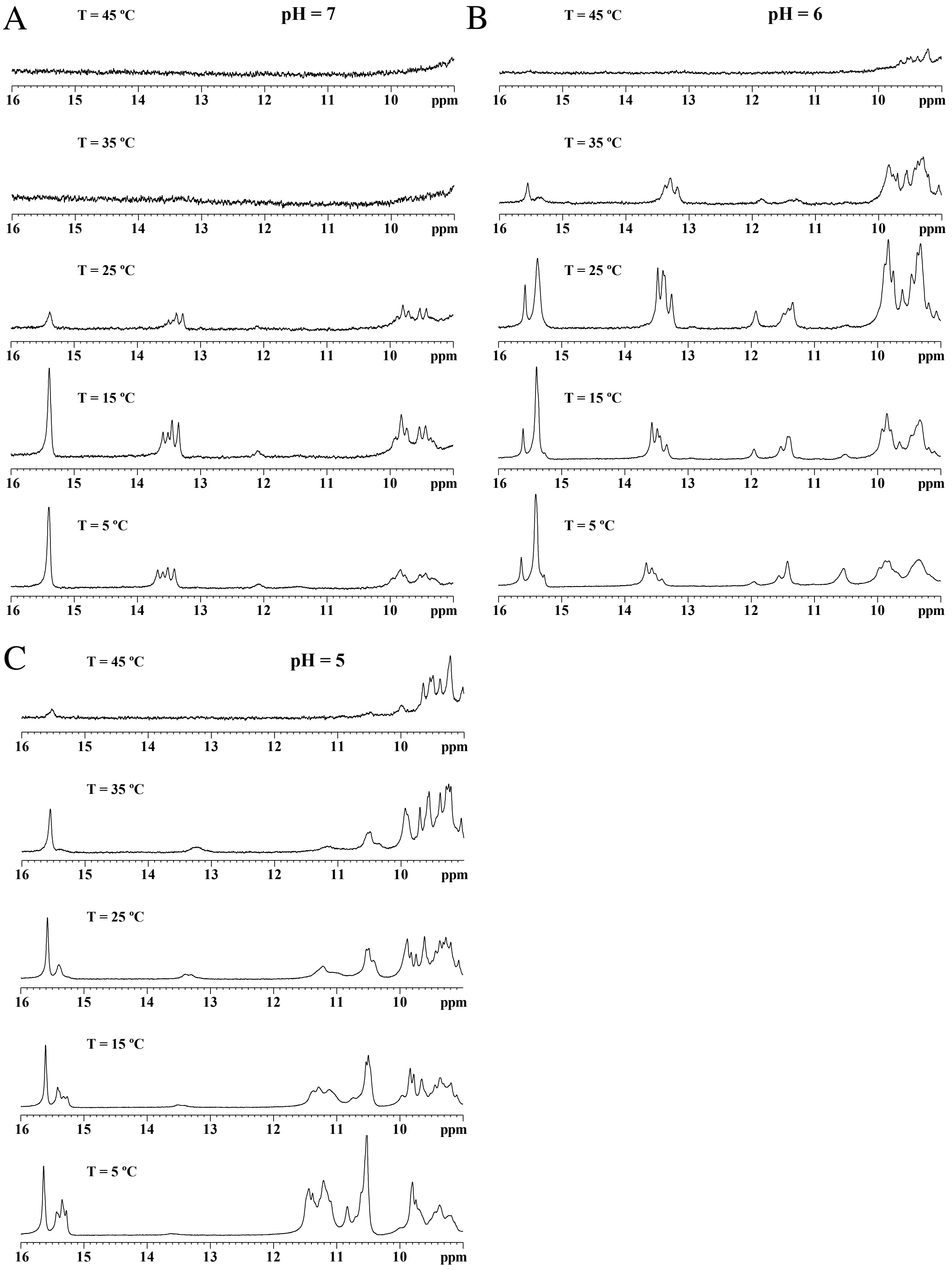

Figure S20.- NMR spectra of and oligonucleotide containing four **N** repeats: **N**-T4-**N**-T4-**N**-T4-**N**, where **N** is d(CCGTTCCGT) at different pHs and temperatures.

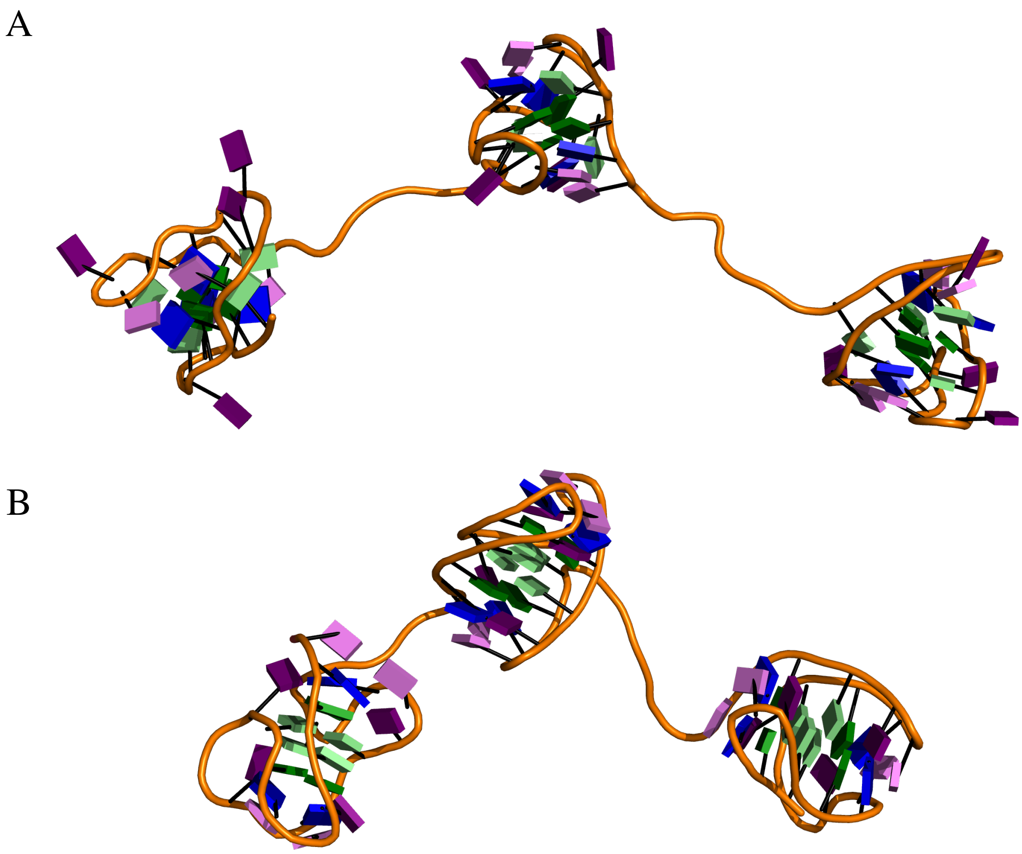

Figure S21.- Models of tandem repeats of **NN4** at neutral (A) and acidic pH (B).

Supplementary Tables

Table S1.- Chemical shifts of **NN4**, pH 7, T=5ºC

	H1/H3/H3$^+$	H42/H22	H41/H21	H6/H8	H5/Me	H1’	H2’	H2”	H3’
C1	-			7.85	6.00	6.23	2.34	2.53	4.83
C2	15.42	9.56	8.16	7.46	6.01	6.23	1.02	2.18	4.68
G3	11.91	8.21	6.53	8.23	-	6.01	2.99	2.68	5.07
T4	n.o.	-	-	7.62	1.74	6.12	2.03	2.34	4.80
T5/T18	n.o.	-	-	7.84	1.94	6.47	2.28	2.56	4.66
C6/C19	-	8.57	7.39	7.91	6.12	6.32	2.46		4.84
C7	15.41	9.76	7.80	7.40	5.86	6.20	0.85	2.08	4.68
G8	13.42	7.95	7.66	8.25	-	6.04	3.03	2.69	5.12
T9	n.o.	-	-	7.61	1.17	5.98	2.03	2.36	4.80
T10[b]	n.o.	-	-	7.63	1.82	6.12	2.41	2.51	4.87
T11[b]	n.o.	-	-	7.70	1.91	6.31	2.40	2.54	4.90
T12[b]	n.o.	-	-	7.73	1.92	6.34	2.38	2.58	4.93
T13[b]	n.o.	-	-	7.74	1.93	6.34	2.37	2.59	4.95
C14	-	n.o.	n.o.	7.90	6.03	6.35	2.42	2.59	4.96
C15	15.41	9.56	8.28	7.52	6.14	6.25	0.99	2.09	4.72
G16	12.30	8.21	n.o.	8.23	-	6.02	3.00	2.69	5.07
T17	n.o.	-	-	7.64	1.79	6.12	2.03	2.34	4.80
C20	15.42	9.76	7.80	7.40	5.86	6.20	0.85	2.08	4.68
G21	13.38	7.92	7.74	8.23	-	6.01	2.99	2.68	5.08
T22	n.o.	-	-	7.65	1.75	5.99	2.05	2.19	4.45

[a] Exchangeable protons of G8. G21. T4 and T17 have not been specifically assigned.
[b] Modified **NN4** sequences containing site-specific substitution of T10-T13 residues by dU have been used for the assignment of thymine loop residues.
n.o.: not observed
n.a.: not assigned

Table S2.- Chemical shifts of **NN4**, pH 5, T=5ºC

	H1/H3/H3$^+$	H42/H22	H41/H21	H6/H8	H5/Me	H1’	H2’	H2”	H3’
C1	15.66	9.41	8.52	7.95	5.98	6.54	2.08	2.55	4.73
C2	15.34	9.67	8.43	7.53	5.84	6.11	1.55	2.38	4.63
G3	10.40	8.63	6.50	8.28	-	5.81	2.88	2.62	5.06
T4	n.o.	-	-	7.62	1.62	5.89	2.21	2.64	n.a.
T5	11.42	-	-	7.79	2.06	6.50	2.39	2.62	4.93
C6	15.65	9.17	8.50	7.93	6.09	6.54	2.01	2.54	4.93
C7	15.29	9.20	7.69	7.54	6.07	6.03	1.46	2.27	4.60
G8	9.80	8.50	6.44	8.17	-	5.98	2.88	2.58	5.11
T9	10.50	-	-	7.62	1.76	6.06	2.04	2.37	4.87
T10	11.22	-	-	7.69	1.93	6.29	2.40	2.55	4.90
T11	n.o.	-	-	7.68	1.88	6.22	2.38	2.53	4.88
T12	n.o.	-	-	7.74	1.93	6.32	2.39	2.57	4.95
T13	11.51	-	-	7.80	1.95	6.39	2.41	2.60	5.00
C14	15.65	9.67	8.50	8.07	5.99	6.54	1.91	2.56	4.88
C15	15.29	9.81	8.71	7.56	5.89	6.10	1.62	2.44	4.60
G16	10.51	8.63	6.16	8.28	-	5.81	2.88	2.62	4.88
T17	n.o.	-	-	7.59	1.60	5.84	2.22	2.64	4.83
T18	11.49	-	-	7.77	2.06	6.48	2.40	2.63	4.93
C19	15.66	9.33	8.46	7.98	6.10	6.61	2.14	2.51	4.93
C20	15.34	9.44	7.78	7.57	6.05	6.05	1.70	2.29	4.72
G21	9.78	8.50	6.44	8.19	-	5.94	2.86	2.57	5.08
T22	10.61	-	-	7.65	1.75	5.95	2.09	2.25	4.46

n.o.: not observed/n.a.: not assigned

Table S3.- Chemical shifts of **NN4-M2**, pH 7, T=5ºC

	H1/H3/H3⁺	H42/H22	H41/H21	H6/H8	H5/Me	H1’	H2’	H2”	H3’	H4’
C1	-	n.o.	n.o.	7.93	6.09	6.30	2.37	2.60	4.85	4.23
mC2	15.37	9.76	7.56	7.38	1.89	6.25	1.11	2.14	4.74	4.41
G3	11.90	8.23	6.53	8.19	-	5.96	2.96	2.68	5.05	4.70
T4/T17	n.o.	-	-	7.62	1.75	6.13	2.04	2.33	4.79	3.85
T5/T18	n.o.	-	-	7.83	1.93	6.47	2.95	2.56	4.65	4.54
C6	-	8.58	7.40	7.89	6.14	6.29	2.45		4.85	4.35
C7	15.39	10.16	8.10	7.41	5.88	6.21	0.82	2.06	4.69	4.43
G8	13.41	7.90	7.70	8.24	-	6.02	3.02	2.70	5.12	4.69
G21	13.46	7.88	7.72						5.08	4.64
T9	n.o.	-	-	7.60	1.74	5.95	2.06	2.39	4.81	4.17
T10-T13 [a]										
C14	-	n.o.	n.o.	7.90	6.05	6.43	2.41	2.61	4.97	4.43
C15	15.39	9.03	7.73	7.51	6.06	6.24	1.01	2.18	4.68	4.32
G16	11.82	8.24	6.32	8.21	-	6.00	2.96	2.67	5.07	4.67
C19		8.55	7.41	7.91	6.13	6.32	2.45		4.85	4.35
C20	15.37	9.87	7.71	7.36	5.84	6.17	0.80	2.15	4.63	4.36
T22				7.63	1.74	6.00	2.07	2.23	4.47	4.08

[a] T10-T13 residues have not been assigned.
n.o.: not observed/n.a.: not assigned

Table S4.- Chemical shifts of **NN4-M2**, pH 5, T=5ºC

	H1/H3/H3⁺	H42/H22	H41/H21	H6/H8	H5/Me	H1’	H2’	H2”	H3’
C1	15.68	9.43	8.47	7.92	5.97	6.51	2.09	2.54	4.71
mC2	15.26	9.86	7.72	7.23	1.78	6.09	1.53	2.38	4.60
G3	10.25	8.55	6.65	8.26	-	5.78	2.87	2.60	5.06
T4	n.o.	-	-	7.62	1.63	5.95	2.21	2.61	4.85
T5	11.40	-	-	n.a.	n.a.	n.a.	n.a.	n.a.	n.a.
C6	15.62	9.47	8.64	7.94	6.09	6.56	2.06	2.57	4.93
C7	15.28	9.32	7.76	7.54	6.10	6.06	1.42	2.27	4.60
G8	9.78	8.53	6.41	8.16	-	5.96	2.86	2.56	5.10
T9	10.48	-	-	7.60	1.76	6.06	2.04	2.36	4.87
T10-T12	n.a.	n.a.	n.a.	n.a.	n.a.	n.a.	n.a.	n.a.	n.a.
T13	11.61	-	-	7.80	1.93	6.39	2.41	2.59	4.99
C14	15.62	9.22	8.31	8.07	5.97	6.51	1.84	2.56	4.86
C15	15.28	9.69	8.62	7.53	5.89	6.07	1.58	2.41	4.58
G16	10.46	n.a.	n.a.	8.28	-	5.81	2.88	2.62	5.05
T17	n.o.	-	-	7.58	1.60	5.84	2.20	2.64	4.82
T18	11.51	-	-	7.76	2.05	6.49	2.41	2.64	4.92
C19	15.68	9.28	8.42	7.98	6.10	6.61	2.13	2.51	4.93
C20	15.26	9.62	7.92	7.57	6.00	6.06	1.71	2.28	4.73
G21	9.87	8.51	n.a.	8.87	-	5.93	2.86	2.55	5.07
T22	10.64	-	-	7.63	1.72	5.97	2.07	2.21	4.41

n.o.: not observed/n.a.: not assigned

Table S5.- Experimental constraints and calculation statistics of **NN4** at pH 7 and pH 5.

<table>
<tr><th></th><th colspan="2">pH 7</th><th colspan="2">pH 5</th></tr>
<tr><th colspan="5">Experimental distance constraints</th></tr>
<tr><td>Total number</td><td colspan="2">151</td><td colspan="2">196</td></tr>
<tr><td>intra-residue</td><td colspan="2">54</td><td colspan="2">69</td></tr>
<tr><td>sequential</td><td colspan="2">59</td><td colspan="2">54</td></tr>
<tr><td>range > 1</td><td colspan="2">38</td><td colspan="2">73</td></tr>
<tr><th colspan="5">RMSD (Å)</th></tr>
<tr><td>all well-defined* bases</td><td colspan="2">0.6 ± 0.2</td><td colspan="2">0.6 ± 0.2</td></tr>
<tr><td>all well-defined* heavy atoms</td><td colspan="2">0.8 ± 0.2</td><td colspan="2">0.8 ± 0.2</td></tr>
<tr><td>backbone</td><td colspan="2">2.5 ± 0.7</td><td colspan="2">1.0 ± 0.2</td></tr>
<tr><td>all heavy atoms</td><td colspan="2">3.4 ± 0.7</td><td colspan="2">1.7 ± 0.4</td></tr>
<tr><td>Residual violations</td><td>Average</td><td>Range</td><td>Average</td><td>Range</td></tr>
<tr><td>Sum of violation (Å)</td><td>1.65</td><td>1.43… 2.00</td><td>2.16</td><td>1.87 … 2.56</td></tr>
<tr><td>Max. violation (Å)</td><td>0.32</td><td>0.22 … 0.35</td><td>0.32</td><td>0.20 … 0.37</td></tr>
<tr><td>NOE energy# (kcal/mol)</td><td>11.0</td><td>10.02 … 11.0</td><td>14.16</td><td>13.19 … 14.98</td></tr>
<tr><td>Total energy (kcal/mol)</td><td>- 1341</td><td>- 1404 … - 1215</td><td>-1541</td><td>-1729 … -1395</td></tr>
<tr><td colspan="3">* All except thymines 5,10,11,12,13,18
K_{NOE} = 20 kcal/(mol·Å^2)</td><td colspan="2">* All except thymines 11,12</td></tr>
</table>

Table S6.- Deoxyribose conformations of **NN4** at neutral and acidic pH according to J-coupling data.

Residue	Sugar conformation	
	pH 7	pH 5
C1	-	North
C2	-	South
G3	South	South
T4	-	South
T5	-	South
C6	-	North
C7	-	South
G8	South	South
T9	-	South
T10	-	South
T11	-	South
T12	-	South
T13	-	South
C14	-	North
C15	-	South
G16	South	South
T17	-	South
T18	-	South
C19	-	North
C20	-	South
G21	South	South
T22	-	South

Table S7.- Average dihedral angles and order parameters of the structure of **NN4** at pH 7.

Residue	Pseudorot.		α		β		γ		δ		ε		ζ		χ	
	Phase	Ampl.	Ave.	OP	Ave.	OP	Ave.	OP	Ave.	OP	Ave.	OP	Ave.	OP	Ave.	OP
**C1**	31	169					34	0.4	140	1.0	-168	1.0			100	1.0
**C2**	44	160	30	0.6	-177	1.0	-48	0.6	147	1.0	-170	1.0	-122	1.0	115	1.0
**G3**	39	155	-76	1.0	179	1.0	53	1.0	141	1.0	-154	1.0	-86	1.0	83	1.0
**T4**	37	168	-71	1.0	167	1.0	57	1.0	150	1.0	-119	0.9	155	1.0	135	1.0
**T5**	40	166	150	0.5	161	0.6	63	0.6	150	1.0	-130	0.9	90	0.7	132	1.0
**C6**	31	165	-60	0.6	179	1.0	72	0.7	142	1.0	-109	0.9	-97	0.6	139	1.0
**C7**	37	167	-64	0.8	168	1.0	48	0.9	147	1.0	-165	1.0	180	0.9	118	1.0
**G8**	36	157	-69	1.0	174	1.0	54	1.0	140	1.0	-150	1.0	-84	1.0	81	1.0
**T9**	36	173	-76	0.9	171	1.0	54	1.0	149	1.0	-139	0.9	156	1.0	137	1.0
**T10**	40	169	-169	0.8	175	1.0	66	0.9	148	1.0	-164	0.9	-172	0.8	114	1.0
**T11**	43	154	-77	0.7	174	1.0	71	0.9	141	1.0	-136	0.9	-83	0.8	126	0.9
**T12**	40	162	-156	0.8	179	1.0	58	1.0	146	1.0	-154	0.9	-168	0.7	132	0.9
**T13**	38	158	-102	0.3	-178	0.9	55	0.7	141	1.0	-146	0.9	-85	1.0	123	0.9
**C14**	25	175	62	0.2	-176	0.9	49	0.7	137	1.0	-155	1.0	-143	0.4	129	1.0
**C15**	41	162	47	0.7	-176	1.0	-55	0.7	148	1.0	-168	1.0	-133	1.0	117	1.0
**G16**	41	156	-71	1.0	174	1.0	52	1.0	143	1.0	-150	1.0	-83	1.0	83	1.0
**T17**	38	169	-69	1.0	166	1.0	58	1.0	150	1.0	-122	0.9	155	1.0	140	1.0
**T18**	40	168	134	0.6	164	0.8	53	0.7	151	1.0	-144	1.0	76	0.8	134	1.0
**C19**	28	158	-66	0.6	173	1.0	70	0.7	135	1.0	-129	0.9	-76	0.3	132	1.0
**C20**	37	167	-24	0.5	176	1.0	11	0.5	147	1.0	-164	1.0	-160	0.9	114	1.0
**G21**	38	163	-69	1.0	171	1.0	52	1.0	144	1.0	-161	1.0	-82	1.0	83	1.0
**T22**	36	167	-69	1.0	162	1.0	55	1.0	148	1.0			149	1.0	140	1.0

Table S8.- Average dihedral angles and order parameters of the structure of **NN4** at pH5.

Residue	Pseudorot.		α		β		γ		δ		ε		ζ		χ	
	Phase	Ampl.	Ave.	OP	Ave.	OP	Ave.	OP	Ave.	OP	Ave.	OP	Ave.	OP	Ave.	OP
**C1**	52	30					52	0.8	90	1.0	-174	1.0			-131	1.0
**C2**	179	35	-65	1.0	-158	1.0	69	1.0	148	1.0	179	0.8	-84	1.0	-89	1.0
**G3**	171	27	-77	0.9	-179	1.0	57	1.0	143	1.0	-108	1.0	-94	0.8	-85	1.0
**T4**	167	32	-83	0.8	163	0.9	75	0.8	144	1.0	-82	1.0	91.4	1.0	-165	1.0
**T5**	170	26	-90	0.6	148	0.8	81	0.7	140	1.0	-122	0.9	107	1.0	-101	1.0
**C6**	53	25	-44	0.3	-179	0.9	97.	0.5	104	1.0	-98	0.8	173	1.0	-145	1.0
**C7**	167	35	-52	0.6	171	0.7	77	0.7	145	1.0	-170	1.0	-148	0.8	-109	1.0
**G8**	142	32	-93	0.7	171	1.0	75	0.8	134	1.0	-90	0.9	-93	1.0	-115	1.0
**T9**	185	23	155	0.1	-171	0.9	124	0.4	136	1.0	-84	1.0	104	0.9	-162	1.0
**T10**	92	23	102	0.8	144	0.7	78	0.8	114	1.0	-115	0.9	103	0.9	-100	1.0
**T11**	146	40	-67	0.3	177	0.9	-156	0.1	137	1.0	-161	0.9	-49	0.8	-125	0.6
**T12**	147	42	104	0.3	174	1.0	74	0.4	140	1.0	-124	0.7	-137	0.3	-100	0.6
**T13**	164	30	169	0.5	-155	0.9	54	1.0	139	1.0	-90	0.9	-141	0.3	-123	1.0
**C14**	44	27	148	0.4	-172	0.9	82	0.6	93	1.0	-165	1.0	-146	0.9	-135	1.0
**C15**	123	27	-66	1.0	-172	1.0	69	1.0	120	1.0	-159	1.0	-78	1.0	-117	1.0
**G16**	160	27	-70	1.0	173	1.0	62	1.0	136	1.0	-83	1.0	-79	1.0	-90	1.00
**T17**	168	30	-95	0.8	168	1.0	69	0.8	137	1.0	-76	1.0	90	1.0	-144	1.0
**T18**	187	27	103	1.0	149	1.0	72	1.0	148	1.0	-96	0.9	96	1.0	-91	1.0
**C19**	54	33	68	0.6	-166	1.0	173	0.7	89	1.0	-172	1.0	174	1.0	-136	1.0
**C20**	164	35	-59	1.0	-171	1.0	73	1.0	143	1.0	-176	1.0	-85	1.0	-101	1.0
**G21**	166	32	-68	0.9	168	0.9	66	0.9	143	1.0	-85	1.0	-88	1.0	-100	1.0
**T22**	167	35	-98	0.8	-178	1.0	52	1.0	147	1.0	-174	1.0	134	0.9	-142	1.0
